# Regio-selectivity prediction with a machine-learned reaction representation and on-the-fly quantum mechanical descriptors†

**DOI:** 10.1039/d0sc04823b

**Published:** 2020-12-22

**Authors:** Yanfei Guan, Connor W. Coley, Haoyang Wu, Duminda Ranasinghe, Esther Heid, Thomas J. Struble, Lagnajit Pattanaik, William H. Green, Klavs F. Jensen

**Affiliations:** Department of Chemical Engineering, Massachusetts Institute of Technology 77 Massachusetts Avenue Cambridge MA 02139 USA whgreen@mit.edu kfjensen@mit.edu

## Abstract

Accurate and rapid evaluation of whether substrates can undergo the desired the transformation is crucial and challenging for both human knowledge and computer predictions. Despite the potential of machine learning in predicting chemical reactivity such as selectivity, popular feature engineering and learning methods are either time-consuming or data-hungry. We introduce a new method that combines machine-learned reaction representation with selected quantum mechanical descriptors to predict regio-selectivity in general substitution reactions. We construct a reactivity descriptor database based on *ab initio* calculations of 130k organic molecules, and train a multi-task constrained model to calculate demanded descriptors on-the-fly. The proposed platform enhances the inter/extra-polated performance for regio-selectivity predictions and enables learning from small datasets with just hundreds of examples. Furthermore, the proposed protocol is demonstrated to be generally applicable to a diverse range of chemical spaces. For three general types of substitution reactions (aromatic C–H functionalization, aromatic C–X substitution, and other substitution reactions) curated from a commercial database, the fusion model achieves 89.7%, 96.7%, and 97.2% top-1 accuracy in predicting the major outcome, respectively, each using 5000 training reactions. Using predicted descriptors, the fusion model is end-to-end, and requires approximately only 70 ms per reaction to predict the selectivity from reaction SMILES strings.

## Abstract 2

Integrating feature learning and on-the-fly feather engineering enables fast and accurate reacitvity predictions using large or small dataset.

## Graphical abstract

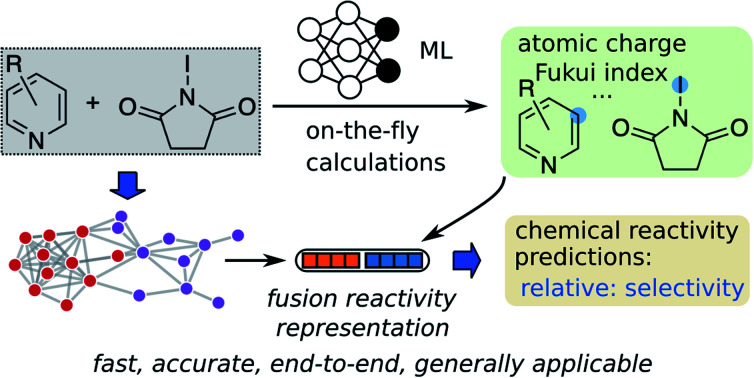

## 1 Introduction

The ability to correctly anticipate chemical reactivity enables chemists to assess whether given substrates might undergo a desired transformation and thus realize the synthesis of a target product more quickly. In this respect, chemical reactivity screening or optimization through automated platforms open the door to the accelerated reaction discovery.^1–6^ Despite many successes in experimental reactivity exploration, fast and accurate *in silico* chemical reactivity modeling (*e.g.* selectivity and yield) remains challenging due to the complex relationship between chemical structures and reactivity.

Quantum mechanical (QM) methods, especially density functional theory (DFT), provide powerful tools to infer reactivity trends of organic reactions, for example *via* the local reactivity descriptors of an individual molecule within the conceptual density functional theory (CDFT).^7–10^ These reactivity descriptors, such as condensed Fukui functions,^11^ indicate how the electron density of a given molecule responds upon the approach of a second reactant, and have been successfully applied to identify the site most prone to either electrophilic or nucleophilic attack.^12–15^ A set of such chemical meaningful descriptors for individual reactants can thus carry key information about chemical reactivity.

**Fig. 1:**
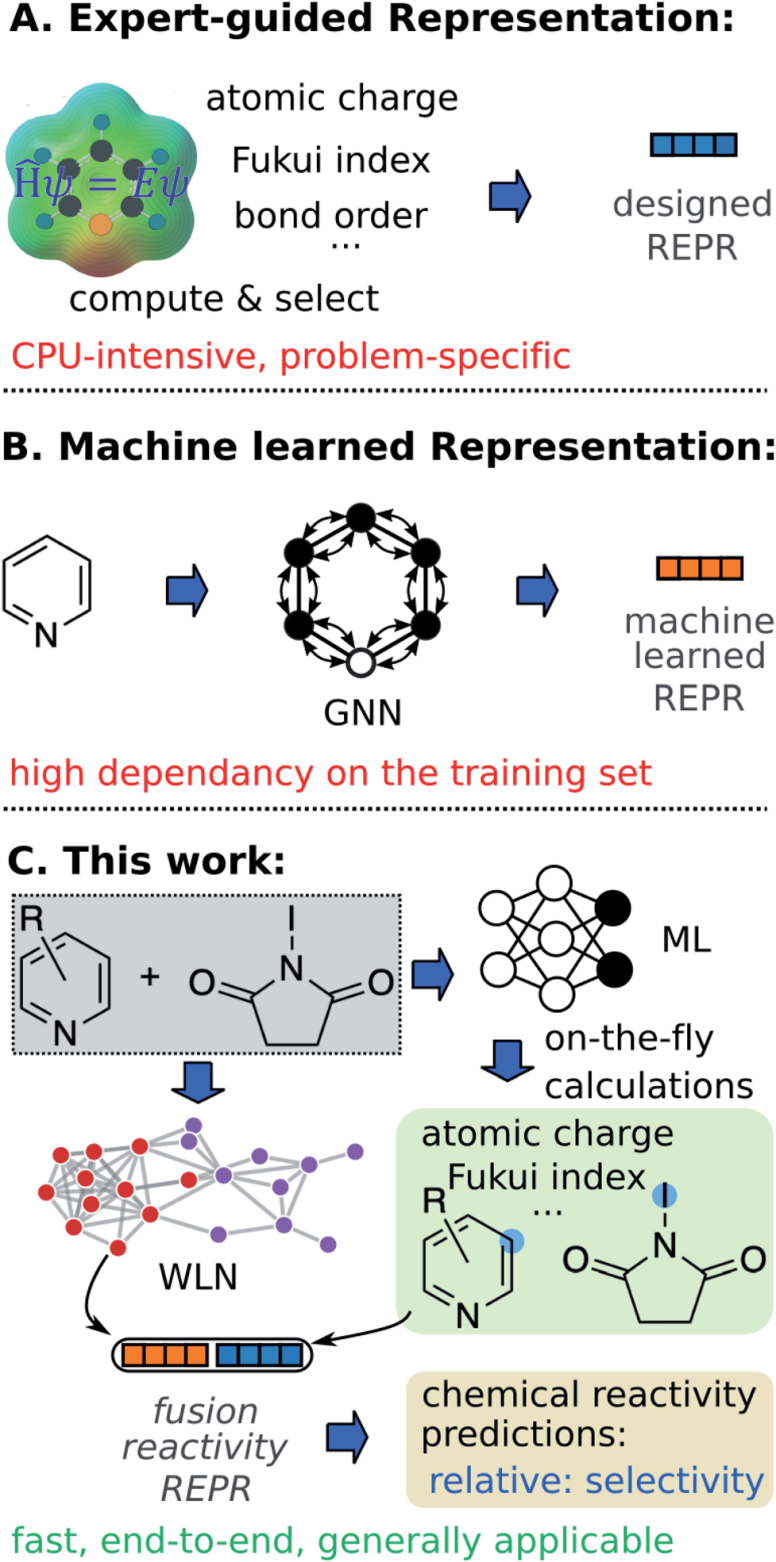
Chemical reactivity predictions using (A) Chemically meaningful descriptors. (B) Machine learned molecular representation. (C) Fusion model with learned reaction representation and on-the-fly calculated quantum mechanical descriptors. REPR: representations.

Machine learning (ML) algorithms, especially feature engineering methods, aim to learn the correlation between a sequence of descriptors and chemical reactivity (Fig. 1A). In the late 1990s, Norrby and co-workers^16,17^ predicted the regio- and stereo-selecitivity for palladium-catalyzed allylation using QSAR and steric descriptors through molecular mechanics. Later works by Lipkowitz and Pradhan^18^ and Melville *et al.*^19^ developed QSSR (quantitative structure–selectivity relationships) methods for predicting enantioselectivity by using comparative molecular field analysis (CoMFA). The recent advance in high-throughput experimentation and data-mining techniques and thus the presence of high-quality data, have significantly populated ML methods in chemical reactivity predictions.^20–28^ Recently, Sigman and co-workers^21,22^ advanced multivariate linear regression to predict the selectivity of a reaction (formally, the difference of free energy barriers), by relying on sophisticated electronic and steric descriptors of substrates and catalysts. An alternate statistical approach built on support vector machines (SVM) and feed-forward neural networks (FFNN) was demonstrated by Denmark and coworkers,^23^ in which the authors proposed a new 3D shape descriptor for catalysts, the average steric occupancy (ASO). Using more than 4000 data points obtained *via* high-throughput experimentation, Doyle and co-workers^24^ demonstrated the prediction of reaction yields of C–N cross-coupling reactions *via* a random forest (RF) model (among other architectures) by selecting reaction-specific descriptors. Although descriptors tailored to a specific reaction class, as seen in feature engineering ML, can be effective representations for predicting chemical reactivity, they might not be generally applicable across reaction and substrate classes.^2^ In other words, such methods are not universal and still require human insight and expertise to design or select corresponding descriptors for each individual task. Moreover, expensive computations associated with QM descriptors often cause bottlenecks in the feature engineering workflow. For example, featurizing a molecule through QM calculations usually requires 3D conformer generation and structure optimization, which usually leads to tedious and time-consuming processes to featurize all molecules in a given dataset.

In addition to expert-guided descriptors, chemical reactivity can be predicted through non-expert descriptors.^29–32^ Typically, reactants and/or reagents are encoded into a 1D vector based on the presence or absence of substructures. Although those structural representations do not carry explicit physicochemical information about molecules, and do not benefit from the insight of experts, the simplicity of this inexpensive fingerprint generation allows fast high-throughput prediction with minimal demands on the user. For example, very recently, Glorius and co-workers^32^ reported success of fingerprint-based ML models in multiple tasks of predicting properties of chemical reactivity.

In feature engineering ML methods described above, reaction representations are built through human intelligence. In contrast, feature learning methods learn representations that capture properties relevant to the prediction task through end-to-end learning (*e.g.* from SMILES strings and 2D/3D structures to properties directly). Compared with other ML models, such feature learning methods including graph neural networks (GNN) and language models have achieved state-of-the-art accuracy in property predictions,^33–37^ and reaction predictions (Fig. 1).^38–44^ With respect to chemical reactivity, GNN models have been demonstrated to be able to predict reaction outcomes given a set of reactants and reagents,^39^ or predict potential electrostatic substitution sites given an aromatic compound.^40^ We note that such feature learning methods usually require considerable training data to offset the lack of functional information in plain molecular graphs or strings to achieve successful end-to-end learning. Furthermore, molecular representations learned from the training set usually show poor out-of-domain performance. Since data deficiency is ubiquitous in the field of chemical reactivity predictions (*i.e.*, often the reactions we most want to predict with are those with the least data), methods that learn well from sparse datasets and exhibit outstanding extrapolation performance are highly desirable.

In the present work, we bridge the gap between feature engineering and feature learning methods discussed above and propose a strategy that unifies the machine learned reaction representation and QM descriptors to predict properties of chemical reactivity, *i.e.*, regio-selectivity (Fig. 1C). We hypothesize that the proposed fusion method could inherit advantages from both feature engineering and feature learning in terms of accuracy, generality, and demand for training data. To overcome the bottleneck of relatively slow QM computations, we construct an *ab initio* database for selected reactivity descriptors and train a multitask neural network to predict QM descriptors for a given molecule on-the-fly. Predicted descriptors are then combined with the machine learned reaction representation to predict regio-selectivity. We note that a number of ML models have been developed to predict some QM descriptors in real time.^37,45–49^ However, to our knowledge, there have not been such a database and model focusing on reactivity descriptors.

We select reactions involving a pair of reacting heavy atoms, such as substitution reactions, to demonstrate the proposed platform. The studied reactions are extracted from a commercial database, Pistachio,^50^ which are more heterogeneous and challenging to predict than the more homogeneous reaction sets obtained through high-throughput experimentation. First, we demonstrate and discuss the fusion model, using QM calculated descriptors, on the task of site-selectivity prediction in electrophilic aromatic substitution (EAS) reactions. A thorough benchmarking shows that machine learned representation and chemically meaningful descriptors complement each other in the fusion model, enhancing performance, and allow learning from a tiny experimental dataset. Second, we implement a multi-task neural network that is trained on DFT calculations of 136k organic molecules to enable on-the-fly calculations for six key atomic/bond descriptors. Finally, we demonstrate the fusion model using on-the-fly descriptors on three general types of substitution reactions including aromatic C–H functionalization, aromatic C–X substitution, and other selective substitution reactions.

## 2 Results

### 2.1 Predicting regioselectivity with machine learned representations and QM descriptors

We start our discussion by implementing the fusion model using machine learned reaction representation and descriptors through QM calculations. First, a dataset containing selective aromatic nitration and halogenation reactions was curated from the Pistachio database *via* reaction templates. Reaction templates were first extracted from selected reactions using RDChiral,^51^ which were then reapplied to enumerate possible products and identify reactions that are site- or regio-selective. The dataset was further filtered to exclude reactions with <50% yield, due to our inability to know with certainty that the reported product in the Pistachio database is the major one and not merely the desired one. In total, 3003 aromatic nitration and halogenation reactions were selected to demonstrate our protocol. Details about the dataset curation and statistics are provided in the ESI S1.2.1 and S1.2.2.†

A graph neural network (GNN), modeled after the Weisfeiler-Lehman network (WLN) architecture for reaction outcome predictions of Jin and Coley,^38,39^ was implemented to predict regio-selectivity. As GNN is a deep data-driven method that is highly dependent on molecular structures it has seen from the training data, it is often challenging to make predictions on structures out of the scope of training set or when training on sparse data. To benefit the model with heuristic information derived from quantum mechanics in addition to the experimental information in the available reaction database, we feed the model quantum mechanical (QM) information of the reactants.

QM methods enable definition of many molecular and local quantities characterizing physicochemical properties of a given molecule. In principle, each such quantity can be employed as a descriptor.^52^ However, many descriptors may carry redundant information. Due to the computational complexities of various descriptors obtained under different levels of theory, covering all accessible descriptors is beyond the scope of this study. In the present work, we focus on a series of the most frequently used local reactivity quantities generated by QM methods, including (1) atomic charges, condensed Fukui functions or Fukui indices,^53^ and shielding constants as atomic descriptors; (2) bond lengths and bond orders as bond descriptors. These descriptors can provide precise quantitative descriptions of electrostatic properties and local environments for each atom. An automated workflow was developed to calculate these descriptors for all reactants starting from a SMILES string. After automated conformer searching *via* Merck Molecular Force Field (MMFF94s),^54^ chemically meaningful descriptors were calculated at the B3LYP/def2svp level of theory.^55–57^ Detailed computational methods are provided in the method section and ESI S1.2.3.†

**Fig. 2:**
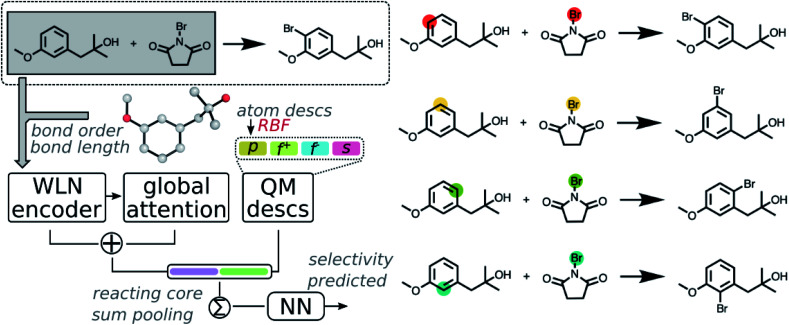
Scheme of the QM-GNN model illustrated on a bromination reaction. Potential reacting centers as well as resulting products are given on the right side. The model starts with a graph-type neural network, WLN, which initializes a feature vector for each heavy atom and bond in the two reactants. The bond is featurized through the RBF expansion of QM computed bond order and bond length. Atom-centered feature vectors are iteratively updated *L* times in the WLN encoder. The updated local atomic embedding is further updated trough a global attention mechanism to capture the influence of atoms further than *L* bonds away, also including atoms on disconnected molecules (*i.e.*, attentions between atoms from the substrate and reagent). Expanded atomic descriptors through RBF are then concatenated to the learned atomic embedding, followed by sum pooling in the reacting core to generate the reaction representation, which finally goes through a dense layer to give the final prediction.

Calculated descriptors were then incorporated into the GNN model to predict site-selectivity. The architecture of the QM enhanced graph neural network (QM-GNN) is shown in Fig. 2. Atomic descriptors and bond descriptors are taken as inputs in different parts of QM-GNN. In the conventional GNN, bonds are often featurized *via* their bond type and ring status. Those discrete bond features are replaced by the continuous bond order and bond length in QM-GNN (in analogy to the 3D SchNet model of Schütt *et al.*^36^), which carry more information than a plain 2D graph. The continuous bond order and bond length were converted into a continuous vector through the radial basis function (RBF) expansion before passed in the WLN encoder. In principle, the atomic features could also be converted from discrete choices of atomic number to continuous QM descriptors. For example, MoleculeNet^34^ and ChemProp^35^ both provide options of using fast calculated empirical descriptors as atomic features. However, our recent studies suggested such a strategy using heuristically-calculated descriptors usually fails to improve the model performance for reactivity predictions.^40^ We feel the critical information carried by QM descriptors could degrade during the message passing due to mixing with other atoms. To best leverage the benefit of QM descriptors, we incorporate atomic QM descriptors only after the WLN encoder and global attention layer have generated the learned atomic embedding, while discrete atom features (including atomic number, degree of connectivity, valence, and aromaticity) are still used as input of the GNN model. The QM atomic features are first expanded *via* RBF expansion and then concatenated with the machine learned atomic representation. The RBF expansion is chosen to ensure the size of the QM descriptor vector matches that of the learned atomic representation thus to prevent the model biasing towards the graph representation. The RBF expansion also serves as a good normalization method for QM descriptors.

Fusion atomic representations combining the graph embedding and QM information are then sum-pooled over reacting atom pairs, *e.g.* the highlighted sp^2^ C and Br atoms in Fig. 2, to represent the reactivity between atom pairs leading to the corresponding major/minor product. We note that in order for the model to automatically determine reacting centers, atom mapping numbers need to be included, which can be obtained through several automatic mapping toolkits.^58–60^ The reacting pair hidden state was then passed through a feed-forward neural network (FFNN) to generate a selectivity score, which is finally scaled to values between zero and one by a softmax function across available products. The softmax function is chosen so that the model is trained to rank major/minor reactions in a relative way. A full description of the model architecture is provided in the ESI S1.3.†

**Fig. 3:**
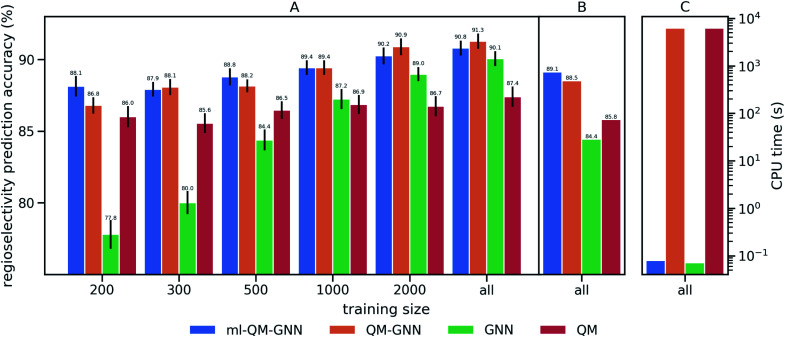
Performance comparison of the QM, GNN, QM-GNN, and ml-QM-GNN models on predicting the regioselectivity of selected EAS reactions. Prediction accuracy indicates the success rate of correctly predicting major products for the testing set. (A) 10-Fold cross-validation based on random splitting of the dataset, where the training set is optionally downsampled to contain only 200, 300, 500, 1000, or 2000 examples for each iteration, while the size of testing set is fixed to 303 for each iteration across different training size. The error bar shows the standard error of the mean of top-1 regioselectivity prediction accuracy on the testing set for each of the 10 cross-validation folds. (B) Performance based on scaffold splitting. (C) Average computation time for predicting the regioselectivity for a given reaction on a single CPU (full training size: *N* = 2397).

We train and evaluate the developed QM-GNN model on the curated EAS reaction dataset for the task of regio-selectivity predictions. The parent models, GNN and QM, were selected as baselines. The GNN model does not use human-specified chemically meaningful descriptors and predicts selectivity based only on machine learned representations. The QM model is a FFNN using QM calculated descriptors for the reacting atoms as input (a full description of baseline models is provided in the ESI†). We chose top-1 success rate of predicting the major reaction to evaluate the model. We first compare the model performance on random splits of data through 10-fold cross-validation. Average values and standard error of the mean of top-1 prediction accuracy for each fold are depicted in Fig. 3A (additional statistics analysis are provided in ESI Fig. S14†).Using all training data, feature learning and semi-feature learning methods including GNN and QM-GNN outperforms the QM feature engineering method (*e.g.* 90.8% *vs.* 87.4% in average prediction accuracy for QM-GNN and QM). To examine the model sensitivity to the size of the training set, we trained models with gradually decreasing training set size, but retain the size of validation and test sets (303 for each fold) so that performance comparisons across different training sizes are based on the same testing examples. As the size of the training set drops down, the accuracy of GNN rapidly declines to 77.8% with 200 training points (an increase of 124% in the error). However, QM and QM-GNN models remain high performing even with a tiny training set (an increase of 11.1% and 29.3% in error for QM and QM-GNN, respectively, with 200 training points). Essentially, QM descriptors carrying more physicochemical information enable a comparatively simpler function to map from descriptors to complex properties.^61,62^ For example, given a set of optimized and expert designed descriptors, even a linear function can predict enantio-selectivity well.^22^ The trend observed above demonstrates that the correlation between QM descriptors and reactivity can be learned even with a training set of just 200 examples. However, since one does not know what the optimized descriptors are for a task *a priori*, the QM model using selected descriptors and relatively simple mapping function is eventually outpaced by the GNN model, as we expose the model to more training examples. Consequently, QM-GNN, the fusion model—inheriting advantages from both QM and GNN—overcomes the limitation of its parent models and achieves superior performance using both the full and reduced training set sizes.

**Fig. 4:**
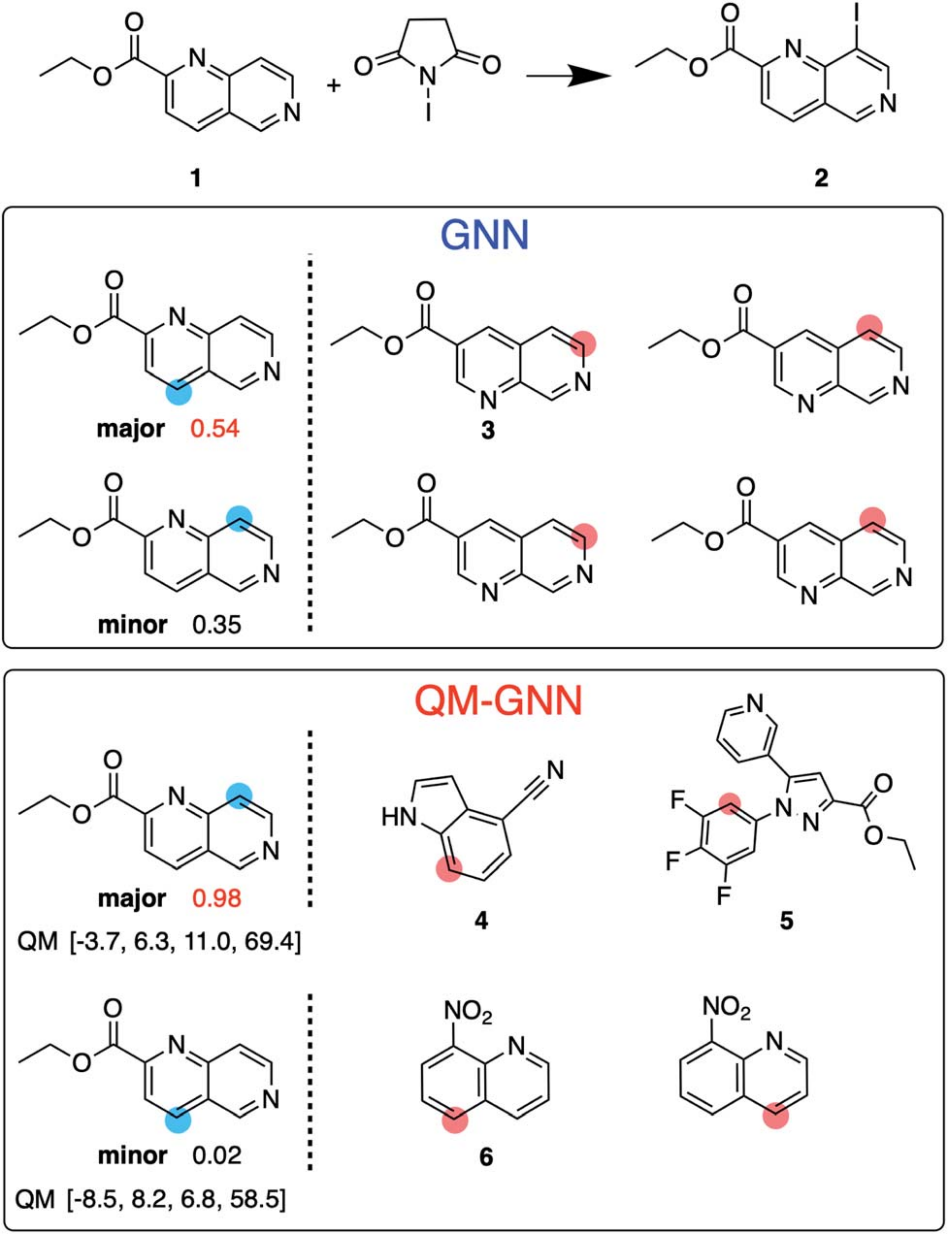
Value of including QM-calculated chemically meaningful descriptors is illustrated using an iodination reaction. Blue dots indicate the major and minor iodination sites. Numbers next to the major/minor site are the predicted score for the selectivity, using GNN or QM-GNN model. The GNN model predicts the wrong major product, assigning reaction to form 2 a lower score (0.35) than formation of the minor product (0.54). The QM-GNN model gives the correct selectivity. For each major/minor site, two most-similar reactions in the training set as judged by each model are given, with the red dot indicating the reacting site. For QM-GNN model, QM descriptors are given for the major/minor site in the order of atomic charge (1e–2 *e*), electrophilic Fukui index (1e–2 *e*), nucleophilic Fukui index (1e–2 *e*), and NMR shielding constant (ppm).

Next, we examine the extrapolated performance of the model *via* scaffold-based splitting. We split the whole dataset based on the scaffold of aromatic rings in a ratio of 80 : 10 : 10 so that training, validating, and testing set do not share common backbones (Fig. 3B). Instead of using cross-validation, we test a single split decided through greedy bin-packing. The scaffold split represents a more challenging evaluation, as molecules in the testing set require a greater degree of extrapolation than in the random split.^61^ Again, the QM-GNN model outperforms GNN (88.5% *vs.* 84.4%). The supplemental QM descriptors implemented in the QM-GNN model facilitate prediction of the out-of-domain unseen examples, which are common in many practical chemical and biochemical problems. In order to further understand the role of QM descriptors in the QM-GNN model, an iodination reaction of compound 1 was selected to study the latent space of GNN and QM-GNN models. The output from the second-to-last layer of the NN in Fig. 2 was extracted from both models as continuous high-dimensional vectors representing two potential reactions (“major” and “minor”). We calculate the Euclidean distance between those two latent vectors and compare it with distances between the major reaction and its neighboring reactions in the training set. Intuitively, for an unseen selective reaction, if the minor reaction is closer to the major reaction than any of its neighbors in the training set, it will be hard to distinguish the two possible outcomes. Here, the distance between the major/minor reactions are similar for the two models (10.7 *vs.* 7.1). However, the neighborhood of the major reaction in the QM-GNN model is far more dense than that in the GNN model (ESI Fig. S16†). The top-2 nearest neighbors of the major/minor reactions in the latent space of two models are shown in Fig. 4. The GNN model tries to distinguish the major/minor outcomes based solely on recognizing structural patterns it learned from the training set (*i.e.*, reactions of compound 3), and thus results in a high overlap between the neighborhood of major/minor reactions (identical top-2 nearest neighbors for major/minor reactions). On the other hand, after incorporating the QM chemically meaningful descriptors, the QM-GNN model is able to look beyond the molecular structure and lead to drastically different neighborhoods for major/minor reactions, which suggests that the QM-GNN model is capable of capturing fundamental physicochemical rules. A statistical analysis on the above trends for the whole testing set is provided in ESI Fig. S16.†

Overall, the QM-GNN model demonstrates outstanding performance in prediction accuracy compared to the conventional GNN and QM models. However, a prominent disadvantage of using QM descriptors is the extra computing time. We can see from Fig. 3C that the computational time for QM-GNN is more than six orders of magnitude larger than that of the GNN model, even with a relatively fast semi-empirical structure optimization method. Calculating the descriptors for a single molecule in QM-GNN took an average of 6200 CPU-seconds. The large computational cost involved impedes the application on large-scale or real-time predictions. In the next section, we describe the development of a deep learning model to rapidly and accurately predict QM descriptors using a multitask and constrained deep learning model, to avoid this CPU-time issue.

### 2.2 Multitask constrained neural network for the fast calculation of QM descriptors

180k organic molecules containing C, H, O, N, P, S, F, Cl, Br, I, Si, B were selected from the ChEMBL^63^ and Pistachio^50^ databases. The automated workflow described in the above section was employed here to perform the high-throughput calculations. About 30% of the initially selected molecules were discarded throughout the workflow, primarily due to imaginary frequencies or timing out. The successful QM calculations on 136k molecules provided a set of more than 26 million data: 4 atomic descriptors (charge, two Fukui indices, NMR shielding constant) for each of 4 363 861 atoms (2 004 079 H atoms and 2 359 782 heavy atoms) plus the bond length and bond order for each of 4 487 376 bonds. More details about data curation are provided in the ESI S1.2.3.†

**Fig. 5:**
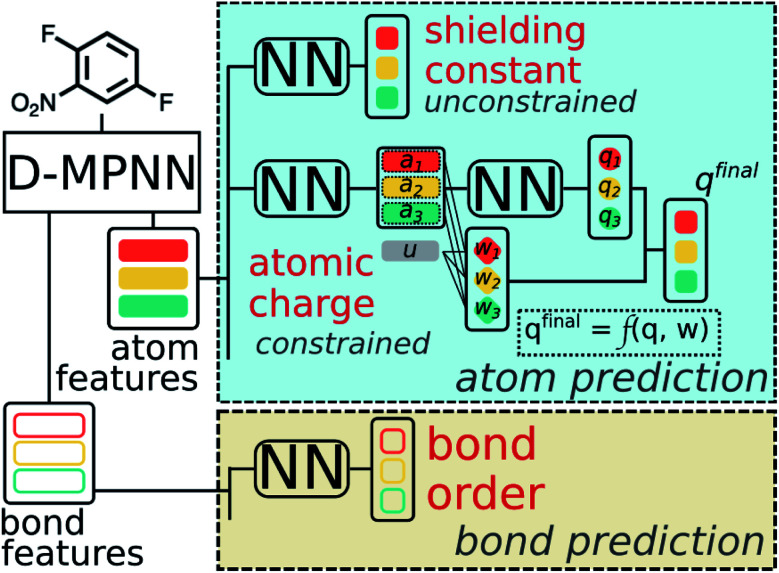
Scheme of the multi-task constrained model to predict chemical meaningful descriptors. The D-MPNN layer, as illustrated in literature,^35^ encodes a molecule into atom features and bond features, which then go through multiple feed forward neural networks (NN) to predict the target descriptors. For constrained descriptors, an attention-based constraint is applied. The constraining function *f* is defined as in eqn (1).

A multitask GNN model was developed to predict multiple QM descriptors from a 2D molecular structure (Fig. 5). The approach was modeled after the directed message passing neural network (D-MPNN).^35^ The D-MPNN encodes a molecular graph into node features and edge features. In principle, we could train a D-MPNN for each type of the chemically meaningful descriptors described above. However, this approach would result in multiple independent models leading to inefficiency and inconvenience in both training and inference. Instead, we constructed a multitask predictor by connecting multiple FFNNs with a single D-MPNN encoder to read out different atomic/bond properties, as shown in Fig. 5. This multitask encoder-readout model uses shared atomic/bond feature vectors as input for different FFNNs, which is inspired by quantum chemical intuition that our target properties, for example partial charges, chemical shift, and bond orders, are derived from the same electronic structure of a given chemical system. Therefore, this synergy is expected to improve the model's ability to learn meaningful functional representations of molecules. Another benefit of the proposed multitask model described above is its ability to systematically handle constraints applied to different atomic descriptors. Taking the atomic charge as an example, a NN is first used to translate the learned atomic representation into initial atomic charges (*q*_*i*_ in Fig. 5). However, throughout the NN, each atomic charge is predicted independently so that the sum of *q*_*i*_ does not necessarily equal the net charge of the molecule. This discrepancy between 
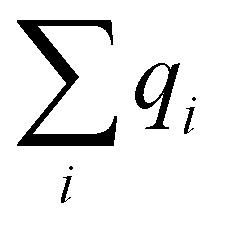
 and the true net charge *Q* can be corrected by spreading the excess charge over the molecule.^64^ Inspired by the word attention mechanism used in natural language processing (NLP),^65^ we developed an attention-based constraining method that determines a weight for each atom to tune how much they need to be corrected. That is, we measure the contribution of each atom to the net correction as the similarity of the atomic hidden representation *a*_*i*_ with a learnable atomic level vector *u* that can be seen as a high level representation of a fixed query “which atom needs more correction?”, and get a normalized weight *w*_*i*_ through a softmax function. The final predicted atomic charge *q*^final^_*i*_ can then be generated from the initial predicted charge *q*_*i*_ and the weight *w*_*i*_ as:1
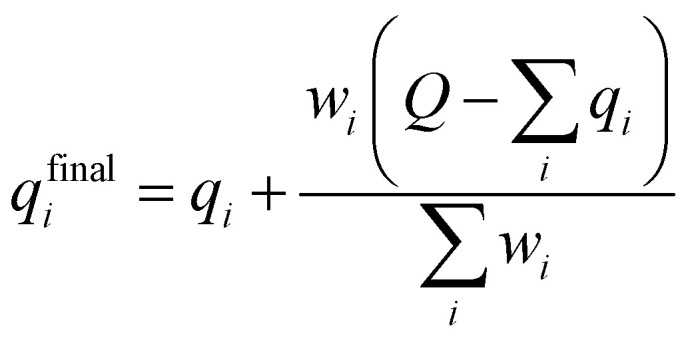


**Fig. 6:**
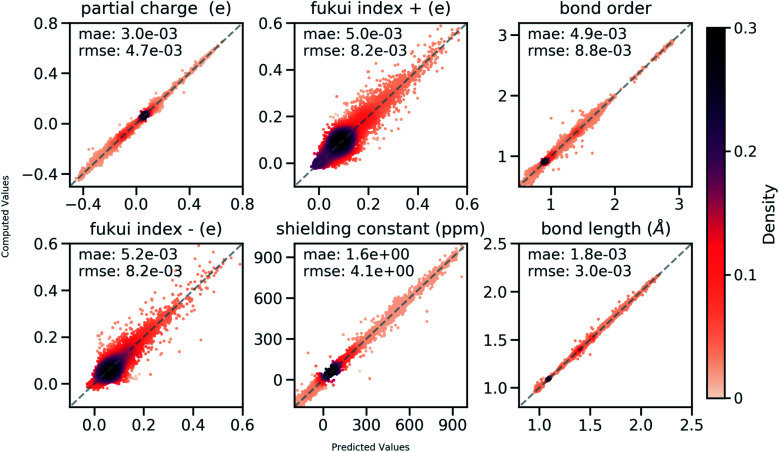
Correlation between QM computed chemical meaningful descriptors and those predicted through the multi-task constrained model on the held-out testing set.

Due to the multitask architecture of our model, the end-to-end constrained learning can be implemented independently for each desired property. All molecules curated are neutral so that the summation constraint is 0 for atomic charges and 1 for nucleophilic/electrophilic Fukui indices, while no constraints are required for NMR shielding constants, bond orders, and bond lengths. Performance of the model was tested on the held-out testing set, consisting of 431 858 atoms and 444 100 bonds. Good correlations between predicted and DFT computed values shown in Fig. 6 suggest that the developed model is reliable in predicting atomic and bond descriptors. More benchmarking studies are provided in ESI S2.3.†

We then use rapidly generated QM descriptors *via* the multi-task constrained model to predict site-selectivity for the 3003 EAS reactions discussed above. We note that compounds involved in those 3003 EAS reactions have been excluded from the 136k training molecules so that the descriptor predicting model does not predict based on memorizing the training data. The prediction accuracy for descriptors of those EAS reactants is similar to the testing set accuracy shown above (ESI S2.3†). The QM calculated descriptors in the QM-GNN model are then replaced by ML predicted ones, referred as ml-QM-GNN. Using the same training and testing methods described above, we evaluate the accuracy and speed of the ml-QM-GNN model on 3003 EAS reactions against the QM-GNN model (Fig. 3). Considering inter/extra-polated behavior, the ml-QM-GNN model maintains a high performance close to the QM-GNN model and significantly outperforms the GNN and QM models. Considering computation time, the ml-QM-GNN model requires only 70 milliseconds to predict the selectivity for a reaction from SMILES strings, which is almost six orders of magnitude faster than the QM-GNN model.

### 2.3 Predicting regioselectivity for general substitution reactions

With the fast and accurate ml-QM-GNN model, we are now able to explore other reaction spaces more efficiently. In this section, we further demonstrate this protocol on more general selective reactions. The ml-QM-GNN model predicts the chemical reactivity using a pair of reacting heavy atoms. Therefore, we extend the present model to all selective substitution and addition reactions involving a pair of approaching heavy atoms, while other types of reactions will be studied in extensions of this work.

**Fig. 7:**
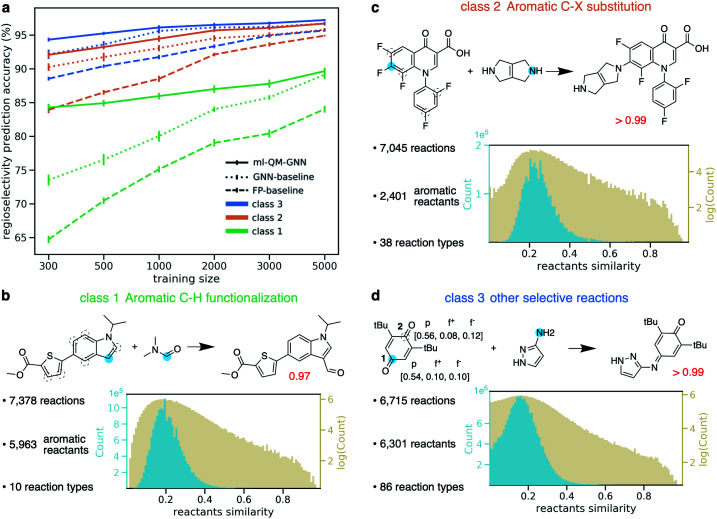
(a) Prediction accuracy comparison of ml-QM-GNN, GNN, and the FP-baseline model in regioselectivity predictions as a function of the training set size for three classes of selective substitution reactions. The error bar shows the standard error of the mean of top-1 regioselectivity prediction accuracy on the testing set for each of the 10 cross-validation folds. Detailed statistics comparison of model performance are provided in ESI Fig. S15.† (b–d) Three classes of selective reactions curated from the Pistachio database along with selected examples. Distribution plots show pairwise Tanimoto similarity between each pair of aromatic substrates (class (1)–(2)) or reactants (class (3)). (d) Blue dots indicate the major reacting site. Dashed circles indicate the minor reacting site(s). The red number shows the predicted selectivity score for major products using ml-QM-GNN. For the example in (d), chemically meaningful descriptors are labeled for major/minor sites in the order of atomic charge, nucleophilic Fukui index, electrophilic Fukui index.

Using the same filtering method discussed above, we extract 20 438 selective reactions from Pistachio, which are further grouped into three classes according to the rough mechanism: (1) 7378 aromatic C–H functionalization (Fig. 7B); (2) 7045 aromatic C–X substitution (Fig. 7C); and (3) 6715 other substitution and addition reactions (Fig. 7D). In contrast to the high-throughput datasets used in pioneering works for descriptor-based chemical reactivity predictions,^22,24,27,32^ reactions curated here are much more heterogeneous in terms of both reaction types and substrate scopes. For example, the 7378 member aromatic C–H functionalization class is composed of 10 types of reactions, involving 5963 unique aromatic substrates and 147 reagents. The pairwise Tanimoto similarity distribution for aromatic substrates shows a single peak at 0.2, indicating the high diversity of molecules studied here (detailed statistics for each reaction class is provided in the ESI S1.2.4†). The ml-QM-GNN model is trained and evaluated on the three curated datasets. 10-Fold cross-validation with gradually downsampled training set and consistent testing set, as discussed above, was applied again to evaluate the model. GNN and a fingerprint-based (FP) model were selected as baseline models here. In the FP-baseline model, the Morgan reaction fingerprint with 2048 bits and a radius of 2, as implemented in RDKit,^66^ was used to encode the major/minor reaction, followed by a FFNN to score the selectivity. This strategy of encoding reactions has been demonstrated to be successful to predict the plausibility of a given reaction on a heterogeneous dataset.^67^ As seen from Fig. 7A, in general, the FP-baseline model showed a poor to medium performance. The ml-QM-GNN model maintains the highest accuracy throughout three classes of reactions. When using 5000 training reactions, the model correctly predicts the major product for 89.7% reactions in class (1); the top-1 accuracy is 96.7% and 97.2% for class (2) and (3), respectively. The ml-QM-GNN model retains a high accuracy with reduced training sets (*e.g.* 84.3%, 92.1%, and 94.3% for classes (1)–(3) when using 300 training points, respectively, which is about half the size of the testing set). The GNN model also achieves a remarkable performance that is comparable to ml-QM-GNN when trained on a large training set. However, the performance of GNN quickly declines as we downsample the training set, especially for the more challenging class 1 dataset.

Selected examples from three reaction classes are also provided in Fig. 7B–D. Selectivity for the class (1) example is mainly driven by the nucleophilicity of competing sites, while the selectivity for the class (2) example is dominated by electrophilicity. The class (3) example is an alkylimino-de-oxo-bisubstitution reaction, which follows the nucleophilic addition mechanism. Regarding proneness toward the nucleophilic attack, the minor reacting site (site 2) is more or at least equivalently likely to react compared to the major site (site 1), as indicated by the Fukui indices in Fig. 7D. The preference for the major site is dominated by the steric hindrance at the minor site from two *t*-Bu groups. The ml-QM-GNN model correctly predicts the major reacting site with high confidence, suggesting that in addition to the electrostatic effect captured by the chemical meaningful descriptors, the machine learned molecular representation implemented is also able to learn the steric effects by recognizing similar structure patterns.

## 3 Conclusion and outlook

This work introduced a novel platform to predict the selectivity of chemical reactions that combines machine-learned reaction representation and quantum mechanical descriptors, including local reactivity descriptors and bond descriptors. The platform leverages the benefits of QM descriptors while minimizing the additional computational cost through the use of an auxiliary multi-task prediction network based on molecular structures alone. A thorough benchmarking on regio-selectivity predictions demonstrates that the fusion model achieves better performance than the conventional graph neural network and descriptor-based feature engineering model in both inter/extra-polated predictions. The fusion model overcomes limitations of feature learning methods and enables learning from a tiny dataset (*e.g.* 200 training points for 300 testing examples). Further latent space analysis reveals that by using chemically meaningful descriptors, the model can learn richer functional representations of reactions in addition to substructural patterns. The combination of learned reaction representation and on-the-fly QM descriptors therefore leads to a fast, end-to-end, generally applicable, and accurate model for chemical reactivity predictions, which is further demonstrated on the prediction of regio-selectivity for three general types of organic reactions. The model achieves 89.7%/96.7%/97.2% top 1 accuracy for aromatic C–H functionalization, aromatic C–X substitution, and other substitution reactions curated from the Pistachio database, within seconds.

In the present work, we have demonstrated the efficacy of combining graph representation and ML predicted QM descriptors in predicting the regio-selectivity for substitution reactions. In addition to ranking relative reactivity (*i.e.*, selectivity), we note that the fusion ml-QM-GNN model can also be adapted to predict quantitative reactivity measures (*e.g.* reaction yields) for a given reaction, requiring minimum modifications. We further evaluated the ml-QM-GNN model on yield predictions including regression and binary classification tasks using both datasets discussed above and high-throughput experimentation data from Doyle and co-workers.^24^ The unbiased ml-QM-GNN model showed comparable performance to expert-guided feature engineering methods on the regression task and provided a measurable improvement over GNN-based and reaction fingerprint-based models on the binary classification task (ESI S2.5†).

The framework presented here still leaves room for improvement. For example, the fusion model performance could be further improved by using higher level of theory to construct the QM descriptors database and including more explicit steric descriptors such as the solvent accessible surface area (SASA). At this stage, on-the-fly QM descriptors calculation in the proposed platform only supports neutral molecules with C, H, O, N, P, S, F, Cl, Br, I, B elements. However, for more general applications, explicit coverage of charged molecules and transition metals in the QM descriptors calculation will significantly improve the performance of the proposed platform. We mention that in contemporary work, Isayev and co-workers^68^ extended the AIMNet model towards open-shell molecules to predict atomic QM descriptors. Compared with the AIMNet model, our model includes less atomic descriptors, but covers essential bond descriptors and is more automated and straightforward to use requiring only the SMILES string of the reactions of interest as input. Both our ml-QM-GNN and the AIMNet model are not able to predict reactivity descriptors for charged molecules, since those require electron structure information for the double-charged species (*e.g.* to compute nucleophilicity Fukui indices for a cation). Including a comprehensive set of reactivity descriptors for charged states of a molecule therefore would be the next challenging step in the real-time QM descriptors computations.

More broadly, this study demonstrates the power of connecting feature engineering and feature learning in addition to providing a useful and convenient tool for chemical reactivity prediction. Future work will look to expand these approaches to reactions involving more complex intermolecular interactions and mechanisms.

## 4 Computational methods

To generate a computational database covering the proposed atomic/bond descriptors, an automated computing workflow was developed. The workflow started by sampling conformers from SMILES strings using the RDKit library,^69^ and the Merck Molecular Force Field (MMFF94s).^54^ The lowest-lying conformer was then optimized at the GFN2-xtb level of theory.^70^ GFN2-xtb is parametrized for all the elements through radon with emphasis on yielding reasonable structures. Since descriptors of interest within this study are more sensitive to molecular structures rather than energetic values, the selected semi-empirical method can provide reliable structures at low computational cost for more than 136k molecules. A variety of convergence checks were performed to ensure the optimization converged to a correct structure, including checks for imaginary frequencies and ensuring that the molecule did not further converge into other species. The final chemically meaningful descriptors were calculated with the B3LYP functional^55,56^ and the def2svp basis set.^57^ All DFT computations were performed using Gaussian 16.^71^ Bond orders were calculated through NBO 6.0.^72^ More details about the descriptor calculation are provided in the ESI.†

The reaction database used in this work is the Pistachio patent database from NextMove (v3.0 released in June 2019). See ESI† for detailed model structures and training procedures. All code used in this work can be found on GitHub (ESI S1.1†)

## Conflicts of interest

There are no conflicts to declare.

## Supplementary Material

SC-012-D0SC04823B-s001
