# Supplementary material for: Regio-selectivity prediction with a machine-learned reaction representation and on-the-fly quantum mechanical descriptors
Source: Chem Sci. 2020 Dec 22;12(6):2198–208. doi: 10.1039/d0sc04823b (PMC8179287; doi:10.1039/d0sc04823b)
Supplement: SC-012-D0SC04823B-s001 [file SC-012-D0SC04823B-s001.pdf]

Electronic Supplementary Material (ESI) for Chemical Science.
This journal is © The Royal Society of Chemistry 2020

# Supporting Information: Regio-selectivity prediction with a machine-learned reaction representation and on-the-fly quantum mechanical descriptors

Yanfei Guan, Connor W. Coley, Haoyang Wu, Duminda Ranasinghe, Esther Heid, Thomas J. Struble, Lagnajit Pattanaik, William H. Green,* and Klavs F. Jensen*

*Department of Chemical Engineering, Massachusetts Institute of Technology, Cambridge, Massachusetts 02139, United States*

E-mail: whgreen@mit.edu; kfjensen@mit.edu

# Contents


**S1 Additional Materials and Methods** **S-3**

S1.1 Code and Data . . . . . . . . . . . . . . . . . . . . . . . . . . . . . . . S-3

S1.2 Data curation . . . . . . . . . . . . . . . . . . . . . . . . . . . . . . . S-4

S1.2.1 Regio-selective reactions curation from Pistachio database . . . . . . S-4

S1.2.2 $3,003$ electrophilic aromatic substitution reactions . . . . . . . . . . . S-4

S1.2.3 $130k$ training molecules for the multitask constrained model for chemically meaningful descriptors predictions . . . . . . . . . . . . . . . . S-8

S1.2.4 Three classes of general selective reactions . . . . . . . . . . . . . . . S-13

S1.3 Machine learning model architecture . . . . . . . . . . . . . . . . . . . . S-19

S1.3.1 **GNN** . . . . . . . . . . . . . . . . . . . . . . . . . . . . . . . . S-19

S1.3.2 **QM-GNN** . . . . . . . . . . . . . . . . . . . . . . . . . . . . . . S-21

S1.3.3 **QM** model . . . . . . . . . . . . . . . . . . . . . . . . . . . . . . S-23

S1.3.4 **ml-QM-GNN** . . . . . . . . . . . . . . . . . . . . . . . . . . . . S-23

S1.3.5 multi-task constrained model to predict chemically meaningful descriptors . . . . . . . . . . . . . . . . . . . . . . . . . . . . . . . . . . S-23

S1.3.6 Fingerprint baseline model . . . . . . . . . . . . . . . . . . . . . . . S-25

S1.4 Training process . . . . . . . . . . . . . . . . . . . . . . . . . . . . . . S-25

S1.4.1 Cross validation . . . . . . . . . . . . . . . . . . . . . . . . . . . . S-25

S1.4.2 Hyperparameter tuning . . . . . . . . . . . . . . . . . . . . . . . . . S-26

**S2 Additional results** **S-29**

S2.1 Cross validation statistics for regio-selectivity predictions . . . . . . . . . . S-29

S2.2 Latent space analysis for the **QM-GNN** and **GNN** model . . . . . . . . . . S-30

S2.3 QM descriptors prediction . . . . . . . . . . . . . . . . . . . . . . . . . S-33

S2.4 Fingerprint random forest model for selectivity prediction . . . . . . . . . . S-36

S2.5 Model modification for yield predictions . . . . . . . . . . . . . . . . . . . S-37

S2.5.1 Yield prediction as a binary classification problem . . . . . . . . . . . S-37
S2.5.2 Yield prediction as a regression problem . . . . . . . . . . . . . . . . . S-39
S2.6 Raw prediction accuracy for cross-validation . . . . . . . . . . . . . . . . . . . S-41
S2.6.1 Raw cross-validation prediction accuracy for Figure 3A . . . . . . . . S-41
S2.6.2 Raw cross-validation prediction accuracy for Figure 7A . . . . . . . . S-42
**References** **S-46**

# S1 Additional Materials and Methods

## S1.1 Code and Data

Machine learning models for reactivity predictions used in this work can be found on the GitHub, `https://github.com/yanfeiguan/reactivity_predictions_substitution`. The workflow for the QM descriptors high-throughput calculations is also make available on the GitHub `https://github.com/yanfeiguan/QM_descriptors_calculation`. The model used to learn and predict atomic/bond QM descriptors can be found at `https://github.com/yanfeiguan/chemprop-atom-bond`. We also provide a more convenient and easier access to the QM descriptors predicting model through PyPI `https://pypi.org/project/qmdesc/`. All data used to implement the quantum mechanical descriptor predicting model are accessible through figshare `https://doi.org/10.6084/m9.figshare.12818702.v1`. We are not able to share reactivity data curated from the Pistachio database, but we provide scripts for data curation. Users with Pistachio license is able to reproduce all reactivity data with the scripts. As a demonstration of our work, we are allowed to share overlapped reactions that can also be found in the USPTO public database (in another word, the USPTO reactions those can be found in our dataset). That part of data is accessible through the github repo.

## S1.2 Data curation

### S1.2.1 Regio-selective reactions curation from Pistachio database

All selective reactions used in this work were selected from Pistachio database[S1] trough template extracting. First, reactions were selected from Pistachio database that only contain organic neutral reactants (*i.e.* C, H, O, N, P, S, F, Cl, Br, I, B). Reaction templates were extracted from the selected reactions using RDChiral,[S2] which were then reapplied to enumerate possible products and identify reactions that are site- or regioselective.

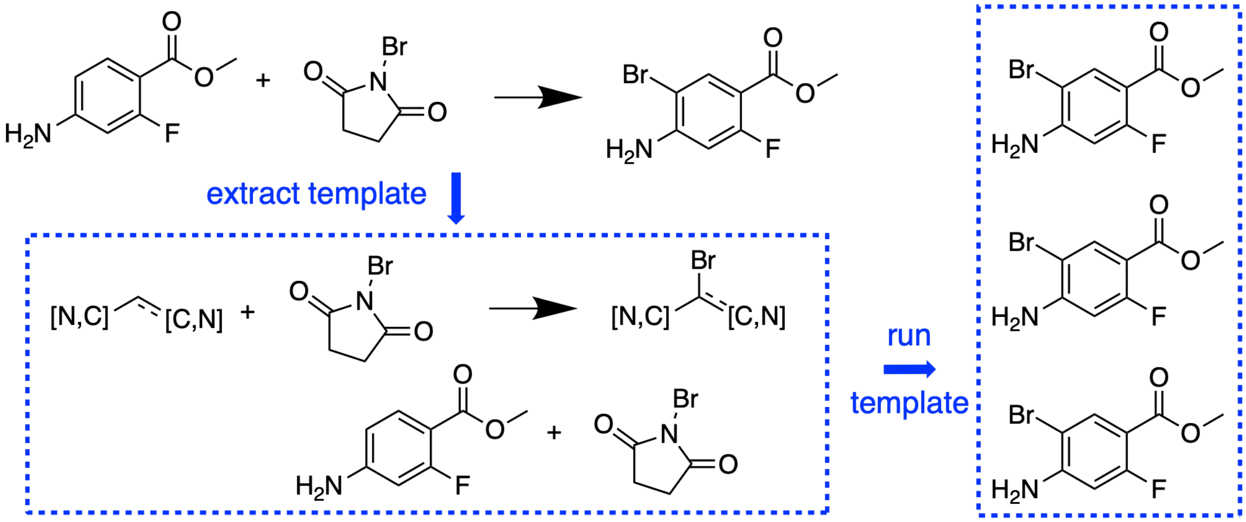


Figure S1: Selective reactions curation from Pistachio database through template applying

### S1.2.2 3,003 electrophilic aromatic substitution reactions

3,003 Electrophilic aromatic substitution(EAS) reactions are composed of Nitration and Halogenation reactions.

Table S1: Description of all reactions included in the 3,003 EAS reactions

| SMILES of other reactants | description | Num. Examples |
|---|---|---|
| O=C1CCC(=O)N1Br | Bromination | 981 |
| O=[N+]([O-])O | Nitration | 791 |

Continued on next page

**Table S1 – continued from previous page**

| SMILES of other reactants | description | Num. Examples |
|---|---|---|
| BrBr | Bromination | 611 |
| O=C1CCC(=O)N1I | Iodination | 368 |
| O=C1CCC(=O)N1Cl | Chlorination | 340 |
| II | Iodination | 99 |
| ClCl | Chlorination | 18 |
| NC(=O)CCC(=O)NBr | Bromination | 14 |
| ClI | Iodination | 9 |
| O=c1[nH]c(=O)n(Br)c(=O)n1Br | Bromination | 8 |
| NC(=O)CCC(=O)NI | Iodination | 8 |
| COc1cc(Br)cnc1N1CCN(C)CC1 | Bromination | 6 |
| BrCCBr | Bromination | 4 |
| ClCCl | Chlorination | 4 |
| CC(=O)NBr | Bromination | 2 |
| C#Cc1cccc(F)c1 | Fluorination | 2 |
| Cn1ncc(Cl)c1-c1csc(C=O)c1 | Chlorination | 2 |
| O=c1n(Cl)c(=O)n(Cl)c(=O)n1Cl | Chlorination | 2 |
| NC(=O)CCC(=O)NCl | Chlorination | 2 |
| FF | Fluorination | 2 |
| C=CCOC(=O)Nc1ccc(F)c(C(=O)OC)c1 | Fluorination | 1 |
| CCCCOCl | Chlorination | 1 |
| Cc1ccc2c(N3CCNCC3)cc(Cl)cc2n1 | Chlorination | 1 |
| Cc1cc(O)c(Br)cc1C | Bromination | 1 |
| Fc1cc(I)c2c(c1)CCN2 | Fluorination | 1 |
| ClN1CCOCC1 | Chlorination | 1 |

Continued on next page

Table S1 – continued from previous page

| SMILES of other reactants | description | Num. Examples |
|---|---|---|
| Clc1ccc(I)cc1Cl | Chlorination | 1 |
| Clc1cn2cc(Cl)c(Cl)cc2n1 | Chlorination | 1 |
| CCNC(=O)NC(=O)CCl | Chlorination | 1 |
| Brc1cc(Br)c(Br)s1 | Bromination | 1 |
| Oc1cc(F)cc(F)c1Br | Fluorination | 1 |
| Fc1ccc2[nH]ccc2c1 | Fluorination | 1 |
| O=C(Cl)c1cccc(Cl)c1 | Chlorination | 1 |
| O=[N+]([O-])c1cc(O)c(Cl)cc1F | Chlorination | 1 |
| O=C1Nc2ccc(F)cc2[C@]12C[C@H]2c1ccc2cn[nH]c2c1 | Fluorination | 1 |
| O=C(OO)c1cccc(Cl)c1 | Chlorination | 1 |
| O=C(O)c1n[nH]cc1Cl | Chlorination | 1 |
| O=C(O)c1cc2ccc(Br)cc2[nH]1 | Bromination | 1 |
| O=C(O)CCCc1ccc(F)cc1 | Fluorination | 1 |
| O=C(Cl)c1ccccc1F | Fluorination | 1 |
| O=C(Cl)c1cccc(Br)c1 | Bromination | 1 |
| Fc1ccc2c(c1)CCNC2 | Fluorination | 1 |
| Nc1ccc(F)cc1 | Fluorination | 1 |
| Nc1cc(Br)cc(Br)c1 | Bromination | 1 |
| NNc1ccc(F)cc1 | Fluorination | 1 |
| NCc1ccc(Br)cc1F | Bromination | 1 |
| Oc1cc(F)c(F)cc1Br | Fluorination | 1 |
| Fc1ccccc1CCI | Fluorination | 1 |
| Fc1ccccc1-c1cc(Cl)ncc1Br | Fluorination | 1 |
| ICCI | Iodination | 1 |

3,003 EAS reactions contain 2,663 aromatic substrate. Statics about pairwise similarity and molecular weight distribution are given below.

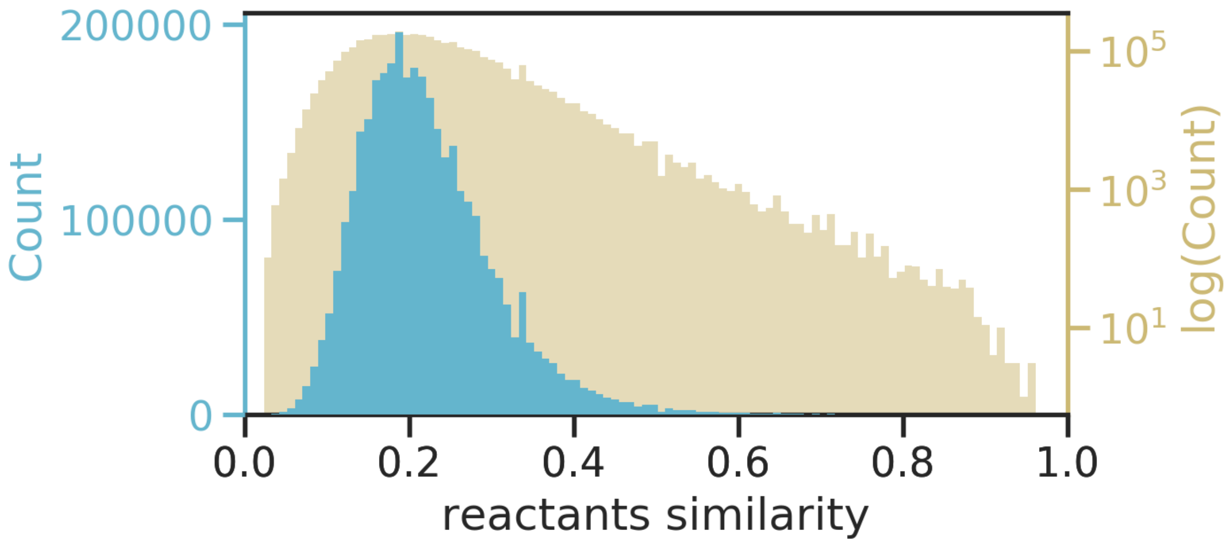


Figure S2: Pairwise Tanimoto similarity distribution between each pair of aromatic reactants in the 3,003 EAS reactions

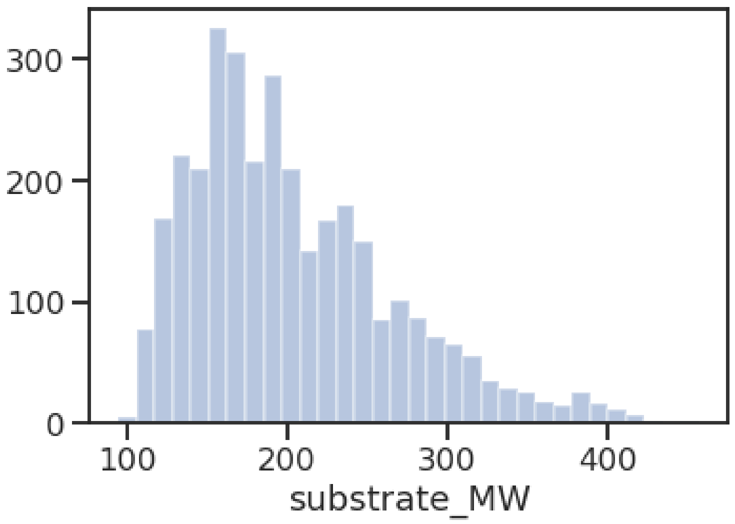


Figure S3: Molecular weight distributions of aromatic reactants in the 3,003 EAS reactions

### S1.2.3 130$k$ training molecules for the multitask constrained model for chemically meaningful descriptors predictions

Training molecules used to develop the multitask constrained model for QM descriptors predictions are curated from ChEMBL and Pistachio. Neutral molecules with molecular weight lower than 500 were selected as follows: 1) For the ChEMBL database, 80$k$ molecules containing C, H, O, N, P, S, F, Cl ,Br, I were randomly selected; 2) for the pistachio database, 100k common reactants, reagents, and solvents that were found in more hant tree reaction records were selected. Due to their abundance in Pistachio dataset, Si and B atom were also included during data extraction, while alkali metal and trainsition metal compounds were discarded considering the complexity and accuracy of the downstreaming computations. Compounds involved in the $3,003$ EAS reactions were then removed. Chemically meaningful descriptors we considered in this work are described below:

The first atomic descriptor we considered is the atomic charge. In the present work, the Hirshfeld partial charge[S3] was chosen as it has a small dependency on basis sets, and are able to reproduce the electrostatic potential, which are important to reactivity and molecular properties.[S4]

Nucleophilicity and electrophilicity play crucial roles in chemical reactivity and many other molecular properties. Condensed Fukui function,[S5] or Fukui indices, by definition, reflects the tendency of each atom towards loosing or accepting an electron, and have been widely used to indicate the neucleophilicity and electrophilicity of single atom. The Fukui indices indicating electrophilicity for atom $i$, $f_i^-$, in a given $N$-electron molecule can be reasonably approximated by the finite-differences method:[S6]

$$f_i^- = q_i(N+1) - q_i(N) \tag{1}$$

where $q_i(N+1)$ and $q_i(N)$ are the partial charge of atom $i$ for the corresponding $(N+1)$- and $(N)$-electron systems with the optimized $N$-electron molecular geometry. Similarly, the

Fukui indices indicating nucleophilicity, $f_i^+$, can be obtained as:

$$f_i^+ = q_i(N) - q_i(N-1) \tag{2}$$

where $q_i(N-1)$ is the partial charge of atom $i$ for the corresponding $(N-1)$-electron systems with the optimized $N$-electron molecular geometry. We note an extra benefit of using Hirshfeld partial charges here that such so-called "stockholders" charge partitioning technique ensures non-negative Fukui indices within the finite-difference approximation.[S7]

Another atomic descriptor we take into consideration is the NMR chemical shift, which indicates the local atomic environment by encoding information about attached or adjacent atoms in the neighborhood and has lead to extensive QSAR/QSPR studies.[S8] In the present work, the NMR chemical shift will be presented as shielding constants and calculated using the Gauge-Independent Atomic Orbital (GIAO) method.[S9]

Bond descriptors considered in the present work are bond length and bond orders. A summary of descriptors used in this work are provided in Table S2:

Table S2: Chemically meaningful descriptors used in this work

| symbol | description | type | tools |
|---|---|---|---|
| $p$ | atomic charge | float | Gaussian16 |
| $f^+$ | nucleophilic Fukui indices | float [0, 1] | Gaussian16 |
| $f^-$ | electrophilic Fukui indices | float [0, 1] | Gaussian16 |
| $sc$ | NMR schielding constants | float | Gaussian16 |
| $bo$ | bond order | float | NBO 6.0 |
| $bl$ | bond length | float | GFN2-xtb |

A workflow was developed using Python to automatically calculate those descriptors in a high-throughput way. The workflow is illustrated in Figure S7

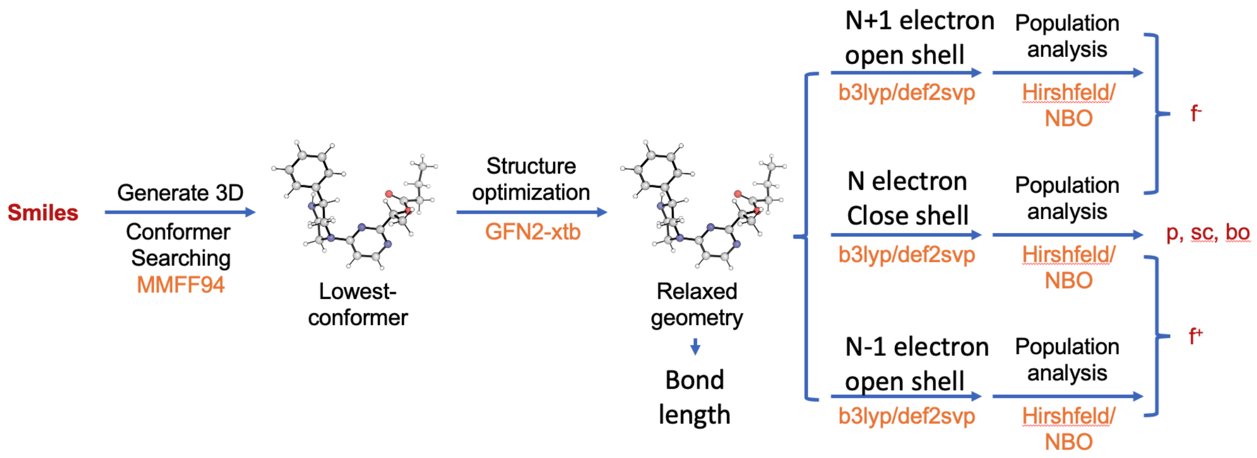


Figure S4: Automated workflow for generating QM descriptors from a given SMILES string. The workflow starts by sampling conformers from SMILES strings using the RDKit library,[S10] and the Merck Molecular Force Field (MMFF94s).[S11] The lowest-lying conformer was then optimized under GFN2-xtb level of theory.[S12] The optimized structure with N, N-1, and N+1 electrons were then sent for DFT calculations under b3lyp/def2svp level of theory. Chemically meaningful descriptors were then obtained through population analysis.

A variety of convergence checks were performed to ensure the optimization converted to a correct structure, including checks for imaginary frequencies and ensuring that the molecule did not further converge into other species, by checking the covalent bond defined in the SMILES string not breaking after optimization. During QM calculations about 30% molecules were discarded due to imaginary frequencies and timing out (14, 400 CPU seconds). Finally, we obtained 136, 219 molecules, including 4, 340, 300 quantities for each atomic descriptors (2, 345, 711 heavy atoms and 1, 994, 589 H atoms), and 4, 463, 203 bond descriptors. All descriptors calculated are available on figshare. Statistics about those training molecules are given below:

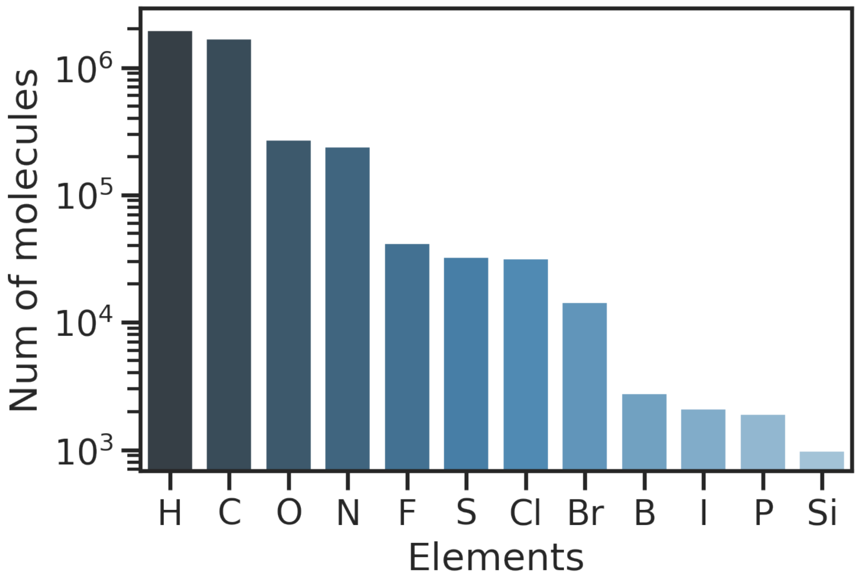


Figure S5: Number of molecules for each type of element in 136$k$ training molecules

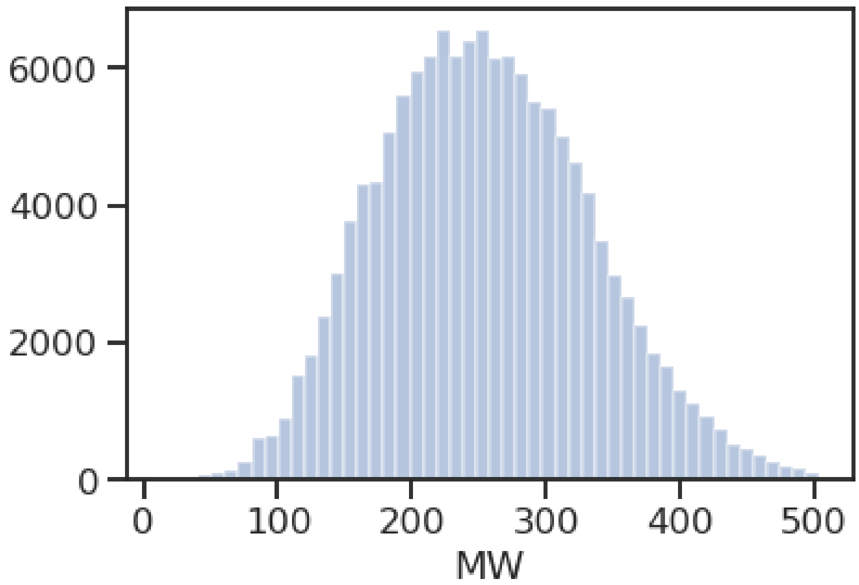


Figure S6: Molecular weight distribution for 136$k$ training molecules

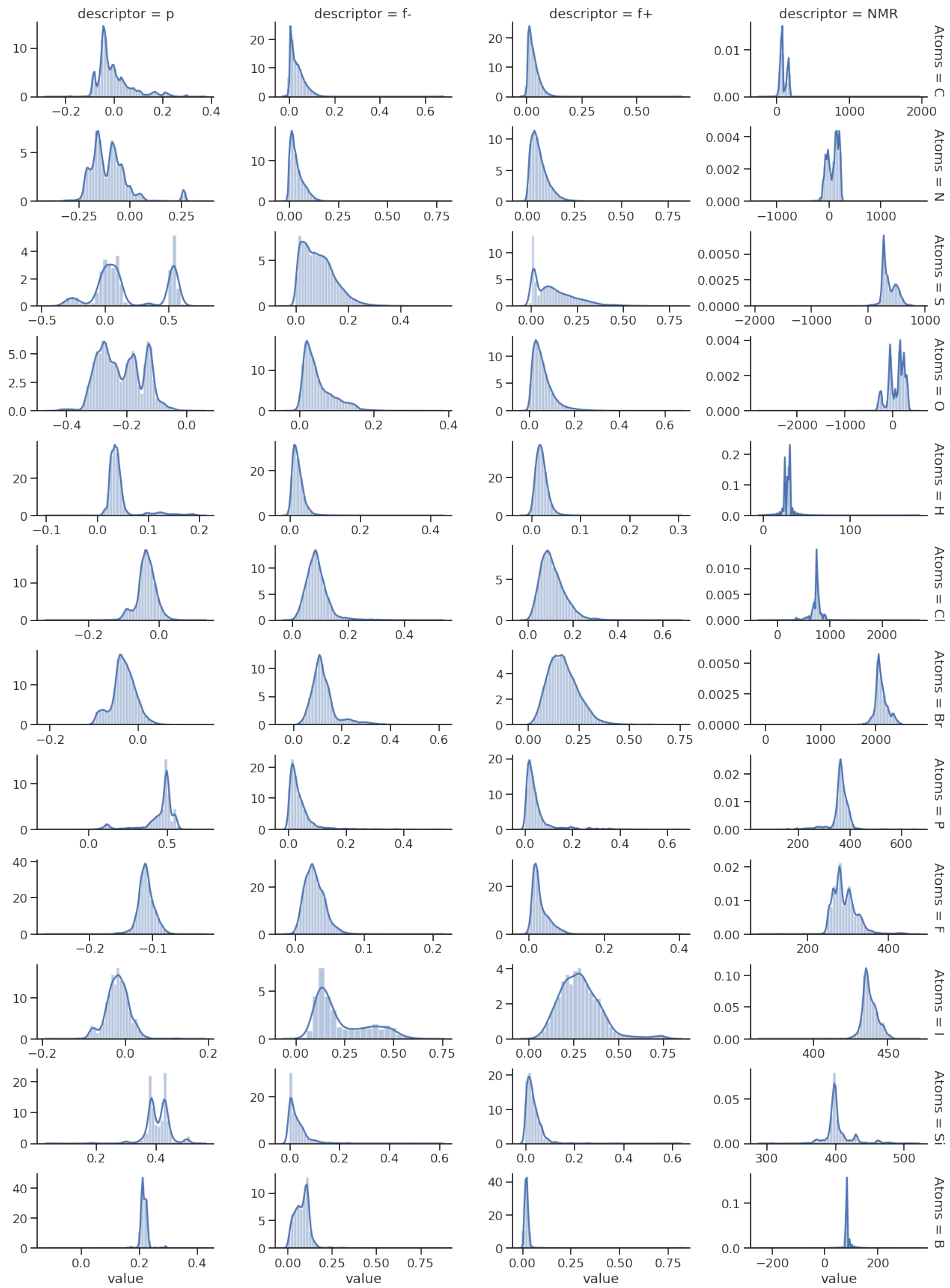


Figure S7: Normalized distribution for each type of descriptors and atoms

### S1.2.4 Three classes of general selective reactions

General selective reactions involving a pair of approaching atoms were selected from the Pistachio database using the same method as depicted in Figure S1, which are further divided into three groups depending on a rough mechanism. Sub-classes involved in each reaction group are given in Table S3–S5.

Table S3: Sub-classes for class 1 reactions: aromatic C-H functionalization

| **reaction type** | **Num. Examples** |
|---|---|
| Bromination | 3338 |
| Nitration | 1513 |
| Iodination | 1156 |
| Chlorination | 904 |
| Vilsmeier-Haack reaction | 373 |
| Fluorination | 44 |
| Sulfonation | 22 |
| Friedel-Crafts acylation | 16 |
| Nitrosylation | 10 |
| Chichibabin amination | 2 |

Table S4: Sub-classes for class 2 reactions: aromatic C-X substitution

| **reaction type** | **Num. Examples** |
|---|---|
| Chloro N-arylation | 2731 |
| Fluoro N-arylation | 1687 |
| SNAr ether synthesis | 1555 |

Continued on next page

**Table S4 – continued from previous page**

| reaction type | Num. Examples |
|---|---|
| Bromo N-arylation | 370 |
| Thioether synthesis | 330 |
| Chloro to amino | 110 |
| Fluoro to amino | 48 |
| Iodo N-arylation | 42 |
| Chloro to hydrazino | 30 |
| Chloro to hydroxy | 24 |
| Fluoro to hydrazino | 15 |
| Fluoro to sulfanyl | 10 |
| Fluoro to cyano | 10 |
| Fluoro to azido | 10 |
| Sulfinic acid + fluoride reaction | 9 |
| Bromo to mesyl | 9 |
| Chloro to thiocyanato | 7 |
| Bromo to amino | 6 |
| Sulfinic acid + bromide reaction | 4 |
| Chloro to cyano | 3 |
| Bromo to hydrazino | 3 |
| Chloro to sulfanyl | 3 |
| Decarboxylative coupling | 3 |
| Sulfinic acid + iodide reaction | 3 |
| Fluoro to hydroxy | 3 |
| Iodo to mesyl | 3 |
| Iodo to thiocyanato | 3 |

Continued on next page

**Table S4 – continued from previous page**

| reaction type | Num. Examples |
|---|---|
| Sulfinic acid + chloride reaction | 2 |
| Bromo to cyano | 2 |
| Bromo N-alkylation | 2 |
| S-Thioester synthesis | 1 |
| Fluoro Gabriel alkylation | 1 |
| Fluoro N-alkylation | 1 |
| Chloro to azido | 1 |
| Bromo to thiocyanato | 1 |
| Hydroxy to chloro | 1 |
| Bromo to hydroxy | 1 |
| Iodo to hydrazino | 1 |

Table S5: Sub-classes for class 3 reactions: other selective reactions

| reaction type | Num. Examples |
|---|---|
| Carboxylic acid + amine condensation | 1022 |
| Bromo N-alkylation | 694 |
| Williamson ether synthesis | 630 |
| Amide Schotten-Baumann | 497 |
| Chloro N-arylation | 451 |
| Aldehyde reductive amination | 311 |
| Fluoro N-arylation | 304 |
| Sulfonamide Schotten-Baumann | 275 |

Continued on next page

**Table S5 – continued from previous page**

| reaction type | Num. Examples |
|---|---|
| O-TBS protection | 170 |
| Ester Schotten-Baumann | 167 |
| Sulfonic ester Schotten-Baumann | 142 |
| Chloro N-alkylation | 142 |
| Lithium Bouveault aldehyde synthesis | 118 |
| Aldol condensation | 113 |
| Formaldehyde reductive amination | 111 |
| N-Boc protection | 111 |
| Indole + ketone condensation | 110 |
| Iodo N-alkylation | 83 |
| Thioether synthesis | 80 |
| Wohl-Ziegler bromination | 78 |
| Ketone reductive amination | 74 |
| Weinreb bromo coupling | 65 |
| Bromo N-arylation | 63 |
| O-Ac protection | 62 |
| N-acetylation | 61 |
| SNAr ether synthesis | 55 |
| Grignard Bouveault aldehyde synthesis | 53 |
| Mitsunobu aryl ether synthesis | 47 |
| Wurtz-Fittig coupling | 37 |
| Esterification | 36 |
| Carboxylic ester + amine reaction | 35 |
| Ketone reductive imination | 32 |

Continued on next page

**Table S5 – continued from previous page**

| reaction type | Num. Examples |
|---|---|
| Hydroxy to methoxy | 31 |
| Bromination | 28 |
| Bromo aldehyde Barbier reaction | 27 |
| Alcohol + amine condensation | 26 |
| Iodo aldehyde Barbier reaction | 25 |
| Carboxylic anhydride + amine reaction | 24 |
| Iodo N-methylation | 24 |
| Cyanoalkane alkylation | 23 |
| O-TIPS protection | 22 |
| Bromo ketone Barbier reaction | 22 |
| Mesyloxy N-alkylation | 21 |
| O-MOM protection | 17 |
| Tosyloxy N-alkylation | 16 |
| Weinreb iodo coupling | 16 |
| Iodo ketone Barbier reaction | 14 |
| Formic acid + amine condensation | 13 |
| Iodo N-arylation | 13 |
| N-TFA protection | 10 |
| Oxo to thioxo | 9 |
| N-Fmoc protection | 9 |
| Bouveault aldehyde synthesis | 9 |
| Eschweiler-Clarke methylation | 9 |
| Amination | 7 |
| Sulfanyl to sulfinyl | 7 |

Continued on next page

**Table S5 – continued from previous page**

| reaction type | Num. Examples |
|---|---|
| Triflyloxy N-arylation | 6 |
| Tertiary amine oxidation | 5 |
| Steglich esterification | 4 |
| Hydroxy to triflyloxy | 4 |
| Carboxy ester to carbamoyl | 4 |
| Oxo to hydroxyimino | 4 |
| Bromo Grignard reaction | 4 |
| Alkylimino-de-oxo-bisubsitution | 2 |
| Aldehyde reductive imination | 2 |
| Chloro Gabriel alkylation | 2 |
| Mesyl N-arylation | 2 |
| O-TMS protection | 2 |
| Regitz diazo transfer | 2 |
| Nitration | 2 |
| Iodination | 2 |
| Fischer-Speier esterification | 2 |
| Imidazolecarbonyl to amide | 2 |
| Horner-Wadsworth-Emmons reaction | 1 |
| Wittig olefination | 1 |
| Methyl esterification | 1 |
| Weinreb ketone synthesis | 1 |
| Chlorination | 1 |
| Vilsmeier-Haack reaction | 1 |
| Sulfonic acid + amine reaction | 1 |

Continued on next page

**Table S5 – continued from previous page**

| reaction type | Num. Examples |
|---|---|
| Ethyl esterification | 1 |
| Tosyloxy Kolbe nitrile synthesis | 1 |
| Ether synthesis | 1 |
| Sulfinic acid + iodide reaction | 1 |
| Chloro Grignard reaction | 1 |
| Decarboxylative coupling | 1 |

## S1.3 Machine learning model architecture

### S1.3.1 GNN

The **GNN** model is derived from the Weisfeiler-Lehman (WL) graph kernel.[S13] The architecture is designed to embed the computations inherent in WL graph kernel to learn isomorphism invariant representation of atoms. The atom representation is computed by iteratively augmenting the representation of adjacent atoms. Specifically, each atom $v$ is initialized with a feature vector $f_v$ indicating its atomic number, degree of connectivity, explicit and implicit valence, and aromaticity. Each bond $(u, v)$ is associated with a feature vector $f_{uv}$ indicating its bond type and ring status. In the $t$ iteration, we updated atom representations from $f_v^{t-1}$ to $f_v^t$ as follows:

$$f_v^t = ReLU(U_1(V_1 f_v^{t-1} \odot \sum_{u \in N(v)} ReLU(U_2(W_1 f_u^{t-1} \odot W_2 f_{uv})))) \tag{3}$$

where ReLU is the rectified linear unit, $\odot$ indicates the concatenation operation, and $U_i, V_i, W_i$ are learned matrices. After L iteration, the final local atom representation are computed as

$$c_v = V_1 f_v^L \otimes \sum_{u \in N(v)} W_1 f_u^L \tag{4}$$

where $\otimes$ indicates element-wise multiplication. The atom embedding $c_v$ only encodes local structural patterns, namely atoms and bonds accessible within $L$ steps from atom $v$. To capture distant information (*e.g.* information between two disconnected atoms), the local atom embedding $c_v$ will then pass through an attention layer. Through attention layer, we calculate the attention score of atom $v$ upon atom $z$. The "global" atom representation $\widetilde{c_v}$ of atom $v$ is calculated as the weighted sum of all reactant atoms where the weight for atom pair $(v, z)$, $\alpha_{vz}$, comes from the attention module:

$$\alpha_{vz} = \sigma(Q_1 ReLU(P_1(c_v + c_z) + P_2 b_{vz}) \tag{5}$$

where $\sigma$ indicates the sigmoid activation function, $P_i, Q_i$ are learned matrices. The "global" atom representation $\widetilde{c_v}$ is then calculated as:

$$\widetilde{c_v} = \sum_z \alpha_{vz} c_z \tag{6}$$

The final atomic representation for atom $v$, $\widehat{c_v}$, including both local chemical environment and global information are then calculated as:

$$\widehat{c_v} = ReLU(M(\widetilde{c_v} + c_v)) \tag{7}$$

where $M$ is the learned matrix. The final reaction representation is then calculated through a sum-pooling layer, which sum over the atomic representation of the approaching atoms ($a$ and $b$ in Figure S9). The reaction representation go through a feed forward neural network (FFNN) to give the final scores.

$$s = \tau(O(\widehat{c_a} + \widehat{c_b})) \tag{8}$$

where O is the learned matrix, and $\tau$ is the final activation function, which is a *softmax* across major/minor reactions (*e.g.* the four reactions in Figure S9) for a selectivity prediction task, or a *sigmoid* for the yield binary classification problem.

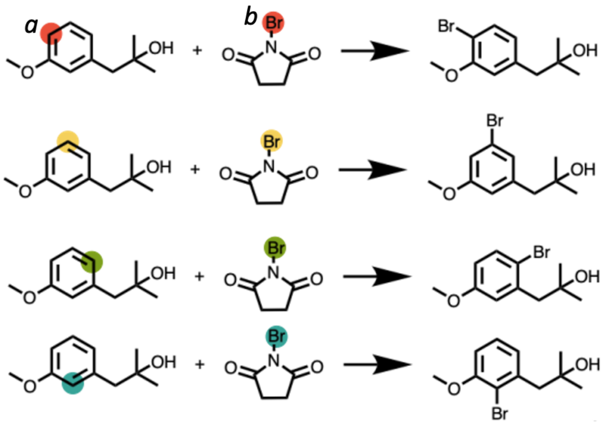


Figure S8: Major and minor reactions for a given selective reactions

### S1.3.2 QM-GNN

Atomic descriptors including atomic charges, Fukui indices, and NMR shielding constants, as well as bond descriptors including bond length and bond order were used as input to the QM-GNN model. First, atomic descriptors are normalized into $[0, 1]$ by min-max normalization. Due to the drastic difference in the scale for NMR shielding constants for different elements, the NMR shielding constant is normalized based on each element type. In the **QM-GNN** model, each bond $(u, v)$ is featurized into $f_{uv}$ through expanding the continuous bond length and bond order via radial basis function (RBF):

$$e_{uv} = [exp(-\frac{(b_{uv} - (\mu + \delta k))^2}{\delta})]_{k \in [0,1,2,...n]} \quad (9)$$

where $b_u v$ is the continuous descriptors (*i.e.* bond length or bond order). $e_{uv}$ indicates the corresponding expanded continuous vecto. $n$ is number of basis functions, which is also the size of $e_{uv}$. $\mu$, $\delta$, and $n$ are thus pre-parameters for the RBF expansions, which are chosen such that the range of the input features are encoded by the centers of these functions. For

example for bond length that ranges from 0.5 to 2.5, $\mu, \delta, n$ are chosen to be $0.5, 0.05, 40$, respectively, which expand the bond length into a vector of size 40 with an interval of 0.05Å starting from 0.5Å. The initial bond vector $f_{uv}$ is then calculated as:

$$f_{uv} = e^{bl}_{uv} \odot e^{bo}_{uv} \tag{10}$$

where $e^{bl}_{uv}$ and $e^{bo}_{uv}$ are expanded continuous vector for bond length and bond order, respectively.

The learned atomic representation is then obtained through the same WLN encoder and global attention mechanism as in the **GNN** model. Atomic descriptors including atomic charge, Fukui indices, and NMR shielding constants are then expanded through RBF expansion and concatenated to the learned atomic representation $\widehat{c}_v$ to give the final atomic representation $\widehat{c}_v^{QM-GNN}$

$$\widehat{c}_v^{QM-GNN} = \widehat{c}_v \odot a^{p}_{uv} \odot a^{f+}_{uv} \odot a^{f-}_{uv} \odot a^{sc}_{uv} \tag{11}$$

where $a^{p}_{uv}, a^{f+}_{uv}, a^{f-}_{uv}, a^{sc}_{uv}$ are RBF expanded continuous vector for atomic charge $p$, nucleophilic Fukui index $f^+$, electrophilic Fukui index $f^-$, and NMR shielding constant $sc$, respectively. Which then go through Equation 8 to give the final prediction.

$\mu, \delta, n$ for all the six descriptors are given below:

Table S6: Pre-parameters of RBF expansion for chemically meaningful descriptors

| descriptor | $\mu$ | $\delta$ | $n$ |
|---|---|---|---|
| $p$ | 0.2 | 0.05 | 10 |
| $f^-$ | 0 | 0.02 | 10 |
| $f^+$ | 0 | 0.03 | 10 |
| $sc$ | 0.2 | 0.08 | 10 |
| $bo$ | 0.5 | 0.1 | 25 |
| $bl$ | 0.5 | 0.05 | 40 |

### S1.3.3 QM model

In the **QM** model, only QM calculated descriptors were used to predict the selectivity. For the example reaction given below:

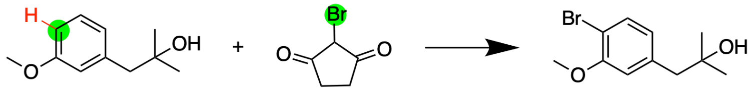


Figure S9: Example of using only **QM** descriptors to predict the reactivity

QM descriptors used as inputs are atomic descriptors $p, f^+, f^-, sc$ for the highlighted atoms and the connected H atom, as well as bond descriptors $bl, bo$ for the corresponding C-H bond. All descriptors are expanded through RBF and concatenated to a single vector of size 185, which is then used as input for a FFNN with three hidden layers (500, 250, and 125 neurons, respectively).

### S1.3.4 ml-QM-GNN

The architecture of **ml-QM-GNN** model is exactly the same as **QM-GNN** model. The only difference lies in that the QM calculated descriptors are replaced by the ML predicted descriptors.

### S1.3.5 multi-task constrained model to predict chemically meaningful descriptors

The multi-task constrained model for the descriptors prediction is composed of two parts: 1) the directed message-passing neural network (D-MPNN) derived from ChemProp.[S14] and 2) the multi-task read-out layers.

The D-MPNN encodes a molecular graph into atom representations and bond representations, which has been illustrated in very detail in the literature. We refer the reader to[S14] for details about the mathematical intuition and justification of the D-MPNN encoder.

The learned atomic/bond representation were then converted into the corresponding

descriptors through a multi-task readout layer. For unconstrained properties, *e.g.* NMR shielding constants, bond order, and bond length, a simple FFNN is used to calculate the descriptor from the feature vector. For the constrained descriptors, such as atomic charges and Fukui indices, we first use a FFNN to calculate the uncorrected descriptors $q_i$ as:

$$q_i = FFNN(\widehat{c_i}) \tag{12}$$

where $\widehat{c_i}$ is the corresponding atomic/bond feature vector. The final corrected descriptor subject to the constraint can then be calculated as:

$$\widehat{a}_i = FFNN(\widehat{c_i}) \tag{13}$$

$$w_i = \frac{exp(u\widehat{a}_i)}{\sum_i exp(u\widehat{a}_i)} \tag{14}$$

$$q_i^{final} = q_i + \frac{w_i(Q - \sum_i q_i)}{\sum_i w_i} \tag{15}$$

where Q is the constraint applied on the descriptor such that:

$$\sum_i q_i^{final} = Q \tag{16}$$

The loss function for each single task optimized the mean absolute error of predicted properties for all atoms or bonds in the molecule. A total loss was then calculated as the weighted sum of each single loss, that brought all losses to approximately the same scale.

$$LOSS = loss_p + loss_{f+} + loss_{f-} + 1e - 5loss_{sc} + loss_{bl} + loss_{bo} \tag{17}$$

where *loss* is the mean squred error (MSE) loss.

### S1.3.6 Fingerprint baseline model

Morgan reaction fingerprint with 1024 bits and a radius of 2 was used to encode the reaction. The reaction fingerprint $fp_{reaction}$ is defined as below:

$$fp_{reaction} = fp_{product} - fp_{reactants} \tag{18}$$

$fp_{product}, fp_{reactants}$ are fingerprints for products and reactants, which are binary vectors with size of 1024. Therefore, the $fp_{reaction}$ is a vector of size 1024 with elements $(-1, 0, 1)$. The reaction fingerprint is then used as input for a FFNN with three hidden layers (500, 250, and 125 neurons, respectively) to give the final prediction.

## S1.4 Training process

### S1.4.1 Cross validation

For selectivity predictions, softmax cross-entropy across major/minor reactions for each set of reactants was selected as the loss function. For the yield binary classification, the sigmoid cross-entropy was selected as the loss function. For the chemically meaningful descriptors prediction, the weighted MSE was selected as loss function.

The 10-fold cross-validation with donwsampling training for the selectivity prediction is performed by first randomly splitting the whole data set into 10 subset. Then the model is trained and evaluated for 10 times. During each iteration, one subset is picked as testing set, and a validating set with the same size as testing set as well as a training set with the corresponding size are randomly sampled from the remaining data. In that way, we guarantee the same testing and validating set for training sets with different sizes.

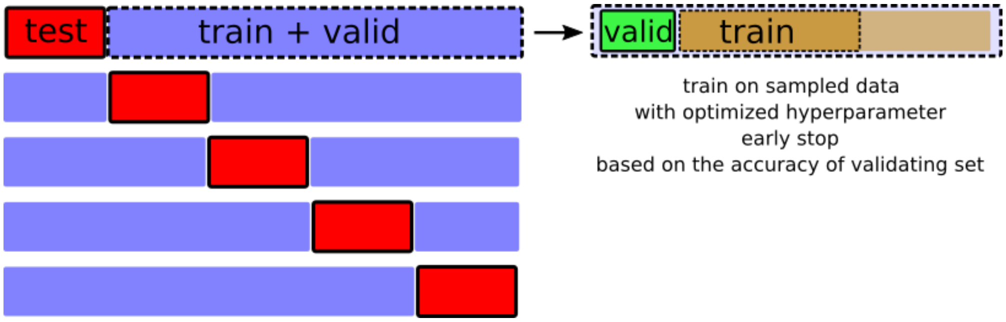


Figure S10: Cross validation used to evaluate models on different size of training sets in the present work. 5-fold cross validation is shown for simplicity

The scaffold-splitting of dataset is based on the Murcko scaffold of the aromatic compounds,[S15] which derives scaffolds of molecule by removing side chains. The whole dataset is splitted into training, validint, and testing set in a ratio of 80 : 10 : 10 using the greedy bin-packing algorithm.

For the 10-fold cross-validation and scaffold splitting experiment, the model was trained for maximum 50 epochs. Early stop based on the loss function on the validating set is employed to prevent over-fitting. The learning rate scheduler was used through out all models. For the selectivity and yield prediction, a reducing learning rate with the decay rate of 0.95 for each epoch was selected. For the multi-task constrained model, a SINEXP learning rate scheduler as defined in the literature[S16] was selected.

### S1.4.2 Hyperparameter tuning

The most appropriate method for the hyperparameter tuning in this case should be the nested cross validation. However, due to the exponential growth in the computational resources required for multiple models, datasets, and training size, we herein use a simplified nested cross-validation (Figure S10).

The hyperparameters are determined through the conventional cross validation method using an example dataset, the EAS regio-selectivity dataset with 1000 training points. The average selectivity accuracy on the validating set is used as the metric.

For **GNN**, **QM-GNN**, and **ml-QM-GNN**, hidden size for the WLN encoder and global

attention layer were determined to be 50. Steps of message passing in the WLN encoder was determined to be 4 (Figure S11).

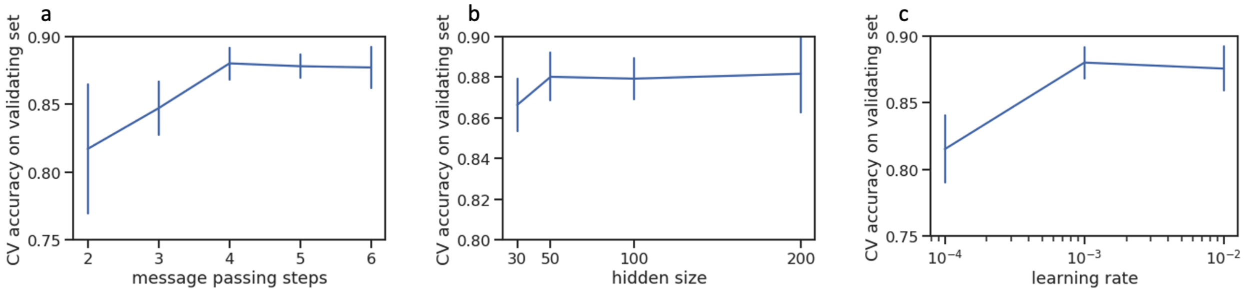


Figure S11: GNN Hyper-parameter tuning for **GNN**, **QM-GNN**, and **ml-QM-GNN** through 10-fold cross validation on the EAS regio-selectivity dataset with 1000 training points. We optimize one hyperparameter at each time and fix other parameters. a) tune the steps of message passing in the graph network. Hidden size is fixed at 50, and learning rate is 0.001. b) tune the size of hidden vectors in the graph network. Steps of message passing is fixed at 4, learning rate is 0.001. c) tune the learning rate. Hidden size is fixed at 50, and steps of message passing is fixed at 4.

.

For the QM model, a trapezoid shaped neural network is used. The number of layers and the size of the first layer was determined to be 4 and 500, respectively (Figure S12).

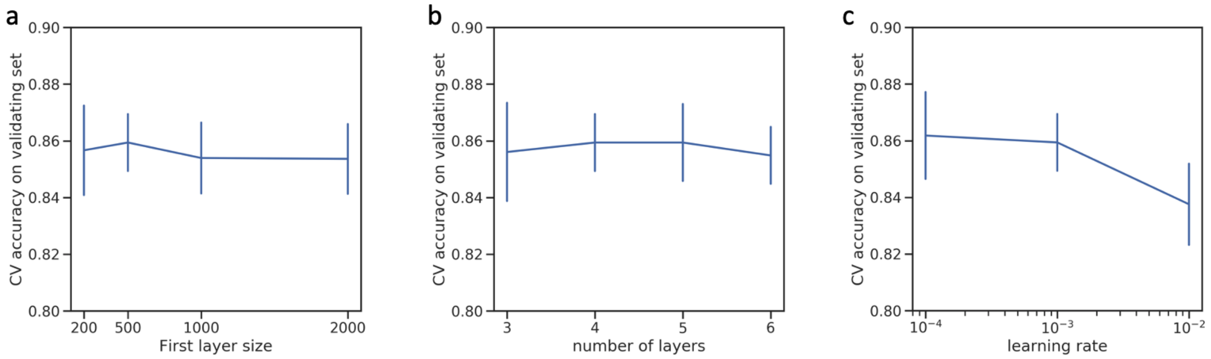


Figure S12: Hyper-parameter tuning for **QM** model through 10-fold cross validation on the EAS regio-selectivity dataset with 1000 training points. We optimize one hyperparameter at each time and fix other parameters. a) tune the first layer size. Number of layer is fixed at 4, and learning rate is 0.001. b) tune the number of layers. Size of the first layer is fixed at 500, and learning rate is 0.001. c) tune the learning rate. Number of layers and size of the first layer is fixed at 4 and 500, respectively.

.

For the fingerprint model, we use a trapezoid shaped neural network as well. Hyperpa-

rameters including number of layers, Morgan fingerprint radius, size of the fingerprint, and learning rate were tuned using the method discussed above (Figure S13).

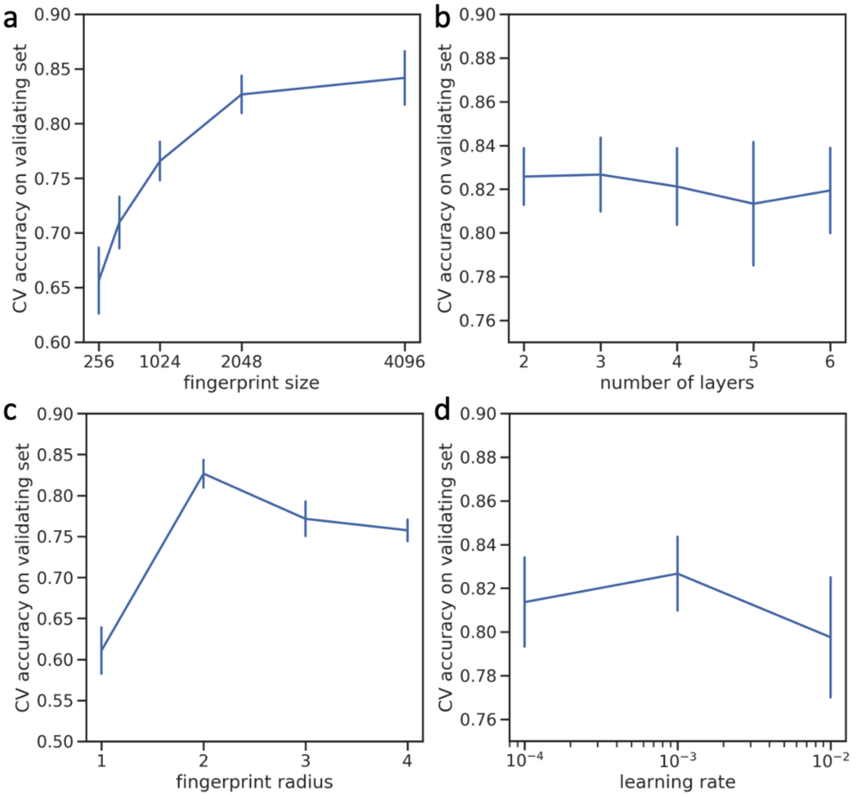


Figure S13: Hyper-parameter tuning for **FP-baseline** model through 10-fold cross validation on the EAS regio-selectivity dataset with 1000 training points. We optimize one hyperparameter at each time and fix other parameters. a) tune the fingerprint size. Number of layers and fingerprint radius are fixed at 3 and 2, respectively. b) tune the number of NN layers. Fingerprint size and radius are fixed at 2048 and 2. c) tune the fingerprint radius. Neural network layers and fingerprint size are fixed at 3 and 2048. d) tune the learning rate.

.

# S2 Additional results

## S2.1 Cross validation statistics for regio-selectivity predictions

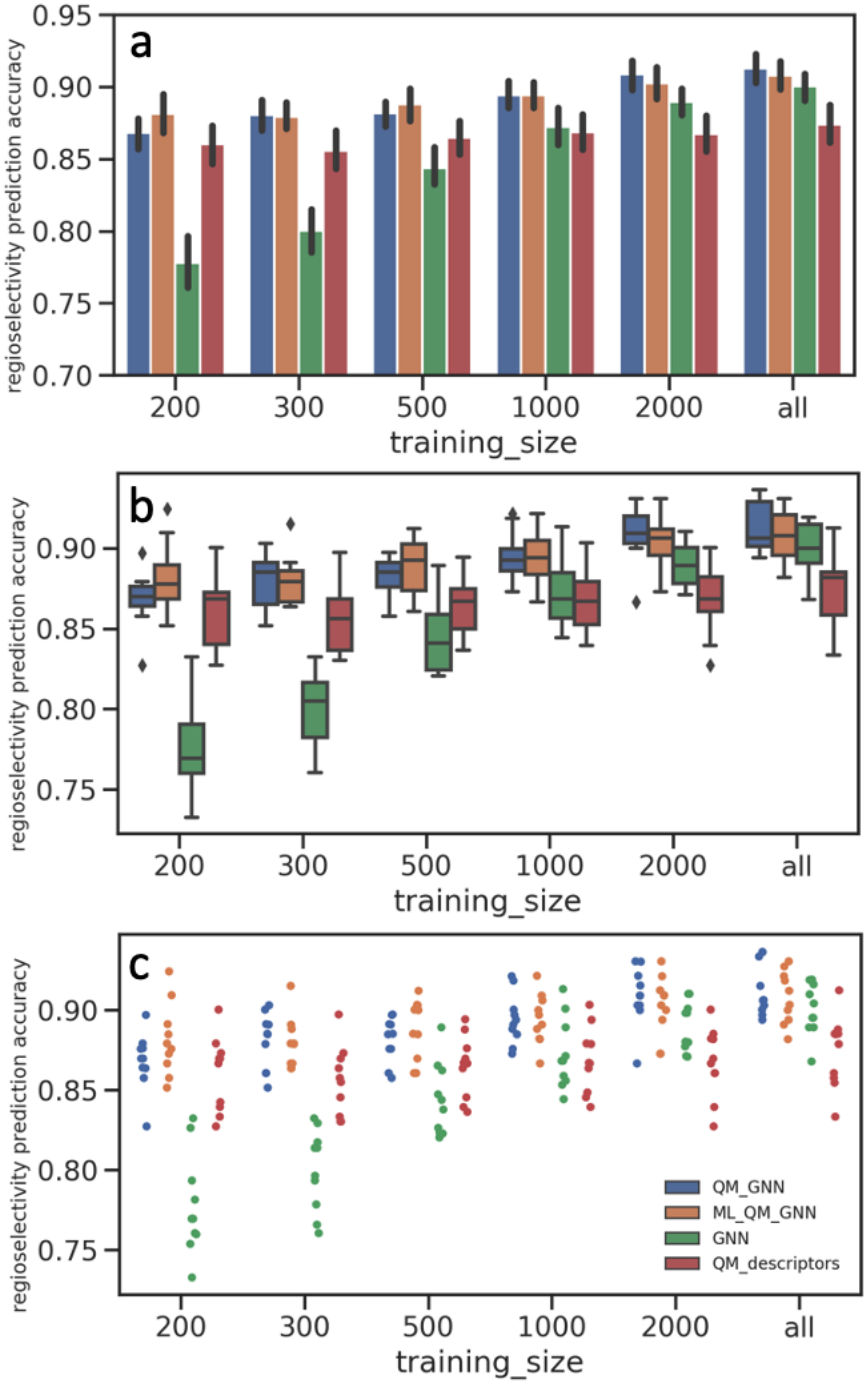


Figure S14: Additional results for the 10-fold cross validation in Figure 3A. a) Error bar shows the 95% confidence interval through the bootstrap method. b) Box plot for regio-selectivity prediction accuracy for each fold. c) Strip plot showing the regio-selectivity predicting accuracy for each fold. Each point stands for the accuracy on the testing set for that fold.

Figure S14 demonstrates: 1) Using a tiny training set, it is significant that **QM-GNN**, **ml-QM-GNN**, and **QM** outperforms the **GNN** model; 2) When we turn to the larger training set end, we are also confident to say that **QM-GNN**, **ml-QM-GNN**, and **GNN** model perform better than the **QM** model.

Similar statistics for three classes of regio-selectivity reactions are provided in Figure S15

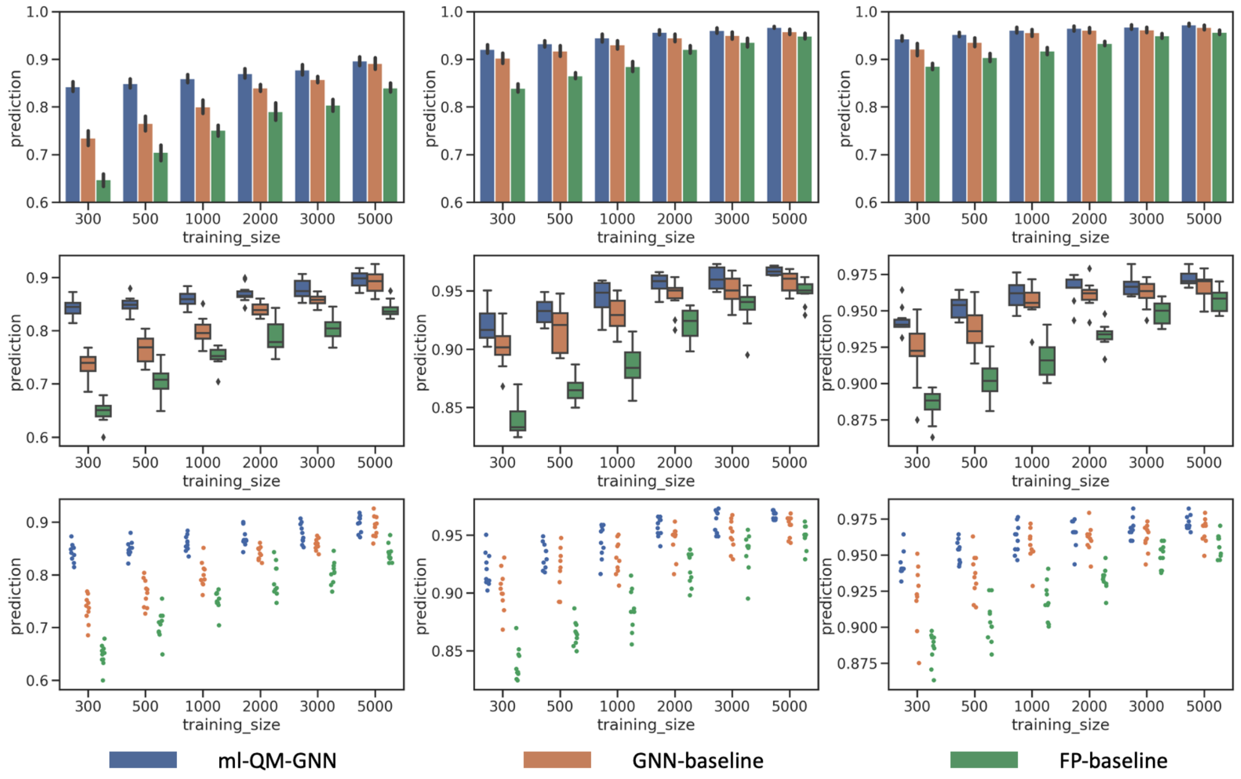


Figure S15: Additional results for the 10-fold cross validation in Figure 7A. First row: Error bar shows the 95% confidence interval through the bootstrap method. Second row: Box plot for regio-selectivity prediction accuracy for each fold. Third row: Strip plot showing the regio-selectivity predicting accuracy for each fold. Each point stands for the accuracy on the testing set for that fold. Left: Class 1 aromatic CH functionalization. Middle: Class 2 aromatic CX substitution. Right: Class 3 other selective reactions.

.

## S2.2 Latent space analysis for the QM-GNN and GNN model

When using a relatively small part of data to train the model (*e.g.* 500 reactions), the **GNN** model incorrectly predicts the major reacting site for 521 reactions during the cross-validation, while the **QM-GNN** model corrects 220 of those making use of chemically mean-

ingful descriptors. Among those corrected reactions, we take the iodination reaction of compound **1** as an example (Figure S17). The selected reaction has five potential outcomes, with **2** as the reported major product and **3** as the **GNN** model's incorrect prediction. The output from the second-to-last layer of the NN in Figure2 was extracted from both models as latent vectors to represent a learned 100-dimensional representation of these two potential reactions ("major" and "minor").

We calculate the Euclidean distance between those two latent vectors and compare it with distances between the major reaction and its neighboring reactions in the training set. Intuitively, for an unseen selective reaction, if the minor reaction is closer to the major reaction than any of its neighbors in the training set, it will be hard to distinguish the two possible outcomes. Here, the distance between the major/minor reactions are similar for the two models (10.7 vs 7.1). However, the neighborhood of the major reaction in the **QM-GNN** model is far more dense than that in the **GNN** model (Figure S17A). The top 3 nearest neighbors of the major/minor reactions in the latent space of two models are shown in Figure S17B. For the selected example, the GNN model, using solely 2D molecular graph information, fails to distinguish the major reaction from the minor reaction. The model has never seen a similar substrate in the training set, and so the major and minor outcomes are significantly closer in feature space to *each other* (a distance of 10.7) than to any examples in the training set (distances of $\geq 77$) . On the other hand, after incorporating the QM chemically meaningful descriptors to supplement 2D molecular representations, we find that there are several training set examples with distances smaller than the distance between the major and minor outcomes (2.3-3.0 are shown versus 7.1). The distribution of distances looks drastically different than the GNN model in both density (cf. Figure S17A) and diversity (cf. structures shown in Figure S17B), suggesting the model is now looking beyond the molecular structure. We then perform a statistic analysis on such trends by counting the number of training points that are closer to the major site than the minor site for each reaction in the testing set (Figure S17C). The distribution supports the trends found for the example

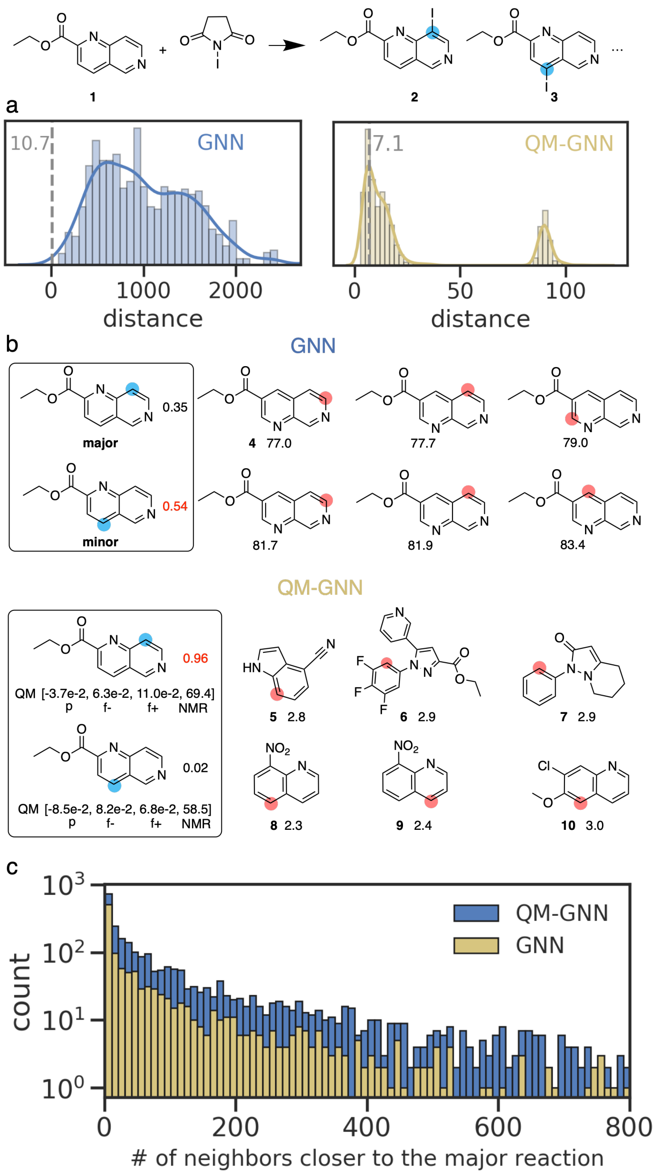


Figure S16: Enhancement of using QM calculated chemically meaningful descriptors is illustrated on an example of iodination reaction. (a) Distribution of distances between the major reaction (leading to **2**) and reactions from the training set. The grey dash line indicates the distance between the major reaction and minor reaction (leading to **3**). (b) Aromatic substrates from the top 3 nearest neighboring reactions in the training set. Blue dots indicate the major and minor reacting sites. Numbers next to the major/minor site are the predicted score for the selectivity. For each major/minor site, three closest neighbors are given, with the red dot indicating the reacting site. Numbers below the nearest neighbors are the distance to the major or minor reacting site of the example reaction. (c) Distribution of number of reactions in the training set that is closer to the major reaction than any of the minor reactions for a given selective reaction in the testing set (0 is omitted for clarity).

reaction. The above discussion indicates that the **QM-GNN** model is capable of capturing the underlying chemical intuitions and learn the fundamental physicochemical rules.

## S2.3 QM descriptors prediction

Error distributions for the descriptor prediction are given below.

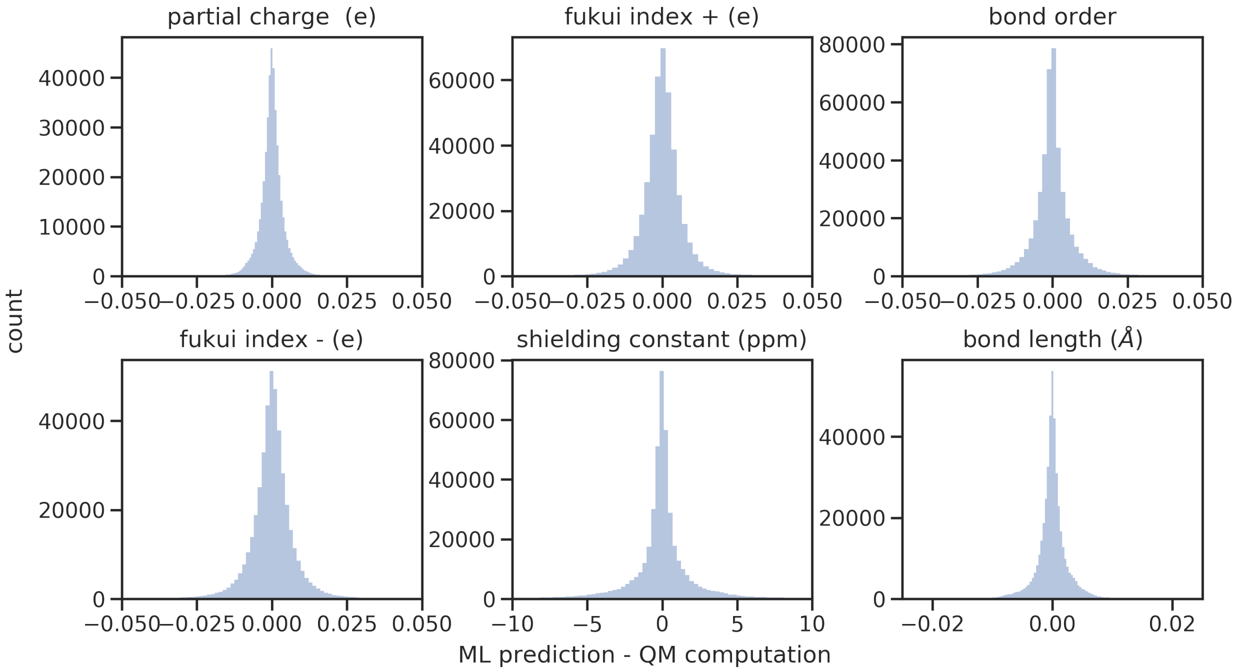


Figure S17: Error distribution on the testing set for the multi-task constrained model.

The correlation of the multi-task prediction shown in Figure 6 varies significantly according to the target, as can be found through $MAE_{scaled}$ in Table S7. Fukui indices are more difficult to learn than others. This is not surprising as the computation of Fukui indices involves an open shell electron structure calculation using the corresponding relaxed structure of the closed-shell species, which introduces larger uncertainty.

We further compared our multi-task constrained model with an atomic fingerprint model developed by Heid *et al.*[S17] (Table S7) as the baseline. In the baseline model, a local fingerprint encoding the local atomic environment such as atoms connected through bonds, angles, and dihedral angles were proposed, which then go through a neural network to give

Table S7: Model and task comparison on the chemically meaningful descriptor predictions

| Properties | This work | | Heid *et al.*[S17] |
|---|---|---|---|
| | MAE | $MAE_{scaled}$[a] | MAE |
| partial charge | **3.0e-3** | 2.5e-3 | 4.0e-3 |
| Fukui index$^{+}$ | **5.2e-3** | 5.8e-3 | 9.6e-3 |
| Fukui index$^{-}$ | **5.0e-3** | 5.9e-3 | 9.3e-3 |
| shielding const | **1.6** | 2.9e-4 | 2.3 |
| bond order | **4.9e-3** | 1.9e-3 | 6.2e-3 |
| bond length | **1.8e-3** | 1.0e-3 | 2.1e-3 |

[a] Scaled MAE as the testing set is scaled to 0-1 by the min-max scaler according to the training set.

the predicted descriptor. Our GNN model outperforms the baseline model across all six targets, especially for Fukui indices. The trend suggests that our model is better capturing delocalized information and thus excels at predicting properties that depend more on global features of the molecule such as the Fukui indices.

For reactants involved in the 3,003 EAS reactions, we predict QM descriptors using the multi-task model and compare the result with the QM calculations.

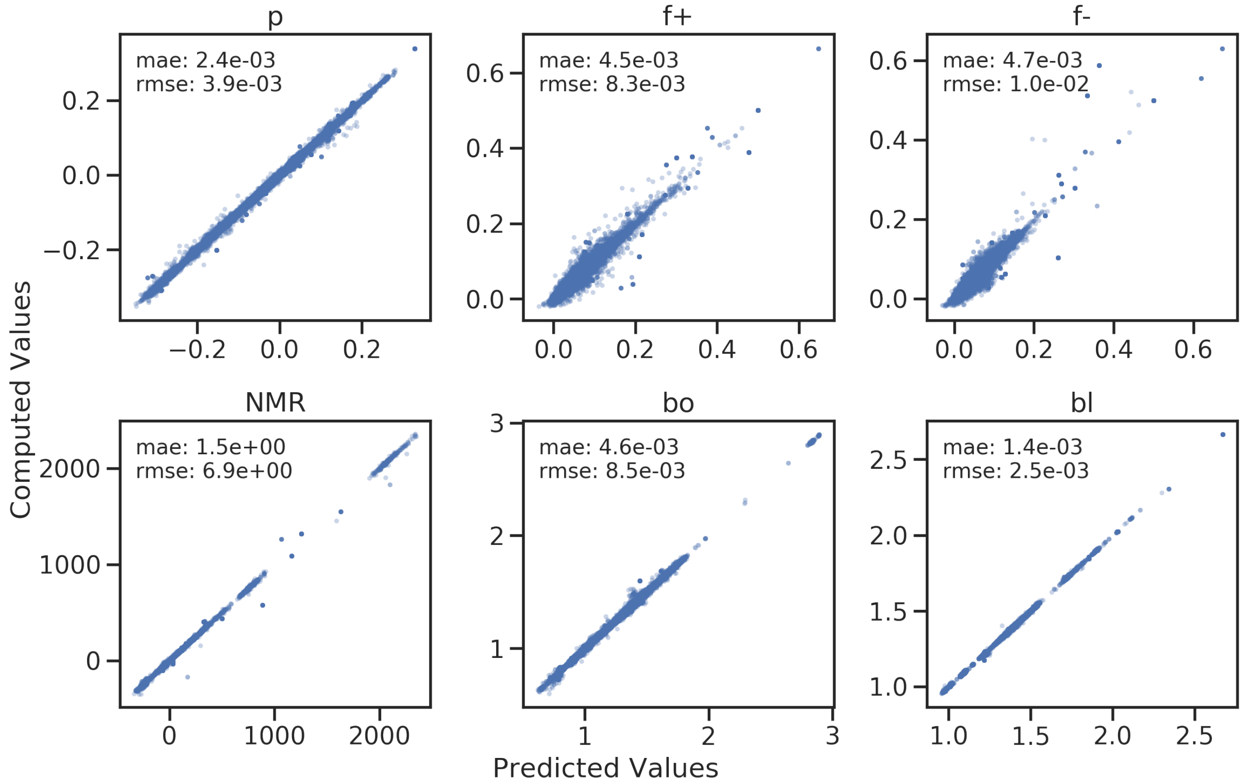


Figure S18: Correlation between QM calculated and ML predicted descriptors for reactants involved in the 3,003 EAS reactions

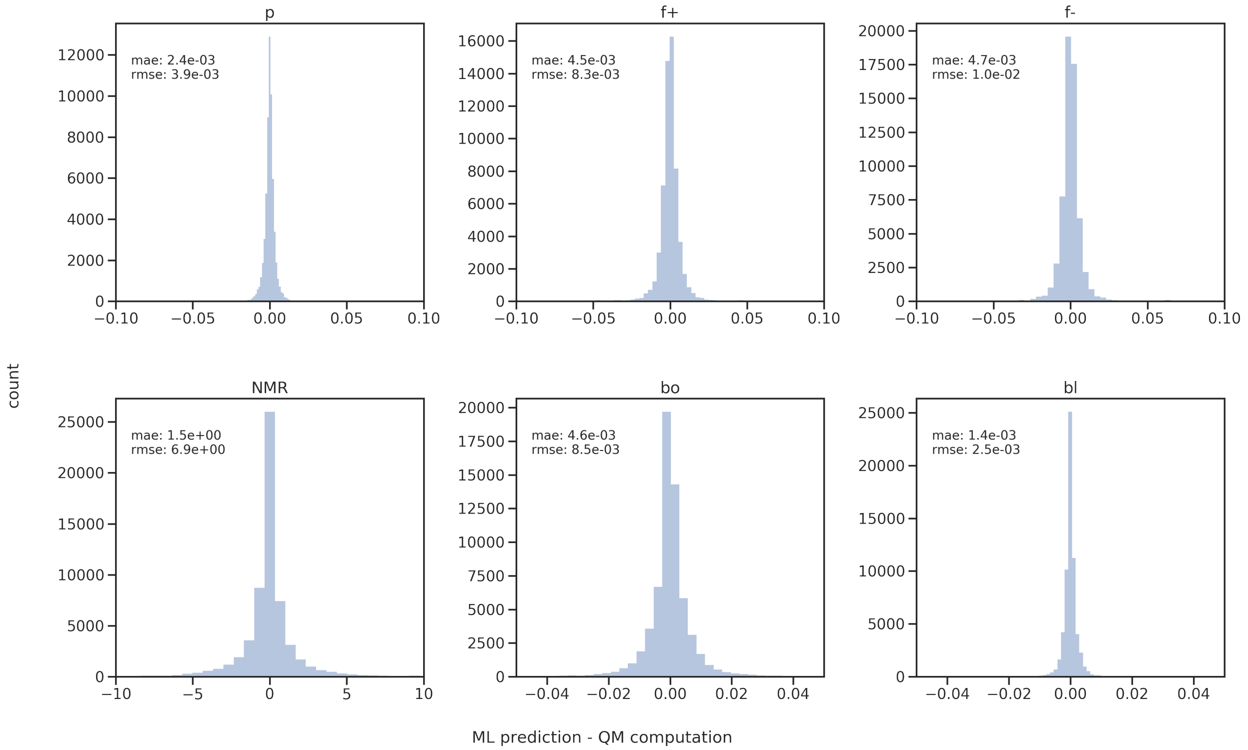


Figure S19: Error distribution for QM calculated and ML predicted descriptors for reactants involved in the 3,003 EAS reactions

## S2.4 Fingerprint random forest model for selectivity prediction

A random forest classifier is trained and evaluated on the tree classes of regio-selectivity reactions. Model hyperparameters are tuned through cross-validation on the 1000 EAS reactions, using random grid search.

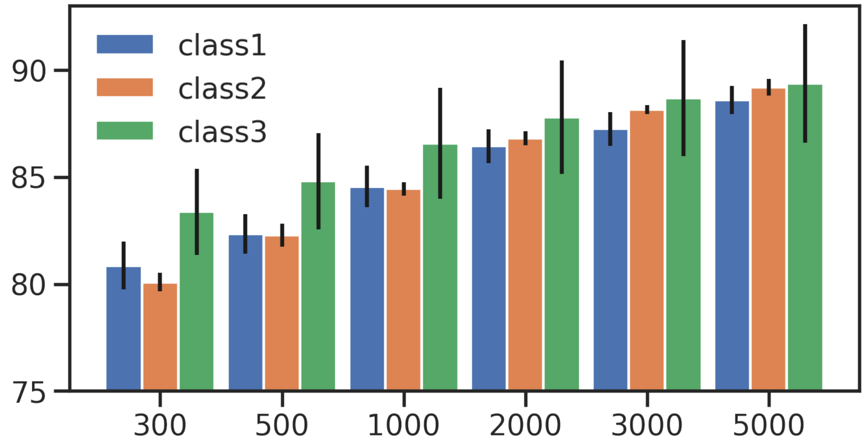


Figure S20: Average cross validation predicting accuracy of random forest classifer model using reaction fingerprint as input to predict the plausibility of each reacting site. The error bar stands for the stand error of the mean of the cross validation.

## S2.5 Model modification for yield predictions

### S2.5.1 Yield prediction as a binary classification problem

The **ml-QM-GNN** model is modified to predict yield for the three general types of substitution reactions studied in this work. Reactions in our dataset are mined from various patent documents so that the recorded yields were not obtained through high-throughput experiments or at consistent conditions. Therefore the yield recorded might be impacted by various effects beyond the actual reactivity (e.g., concentrations, temperature, and reaction times). To account for the fact that the prediction problem is underspecified, but to still attempt to predict the inherent reactivity of reactants, we re-frame quantitative yield prediction into a binary classification problem.[S18]

The yield scale of 0%-100% for reactions studied in the previous sections was divided into three groups. The high reactivity group contains reactions with a yield higher than 70% to guarantee the reaction to have at least a medium reactivity as its inherent property. The low reactivity group is composed of reactions with yields lower than 20%. Reactions with yields between 20% and 70% are discarded due to ambiguity in the actual reactivity. Under such classification, the dataset is dominated by the high-reactivity reactions. To avoid

model biasing toward the high-reactivity end, the low-reactivity group is then augmented by including their minor reactions, which is based on the presumption that the minor reaction of a low-reactivity reaction should also be low-reactive. Subsequently, 8,567 class (1), 9,364 class (2), and 7,226 class (3) reactions containing about 50/50 low/high-reactivity reactions were selected to evaluate the prediction of absolute reactivity (more details are provided in the SI S1.2.5).

Table S8: Model comparison on the task of classifying reactions into low- or high-yielding.

| task | metric | **ml-QM-GNN** | **GNN** | **FP-baseline** |
|---|---|---|---|---|
| class 1 | AUC-ROC[a] | **0.92** | 0.91 | 0.86 |
| | accuracy (%)[b] | **85.6 ± 0.3** | 83.8 ± 0.3 | 78.2 ± 0.4 |
| class 2 | AUC-ROC | **0.89** | 0.88 | 0.86 |
| | accuracy (%) | **82.3 ± 0.4** | 80.9 ± 0.5 | 78.5 ± 0.5 |
| class 3 | AUC-ROC | **0.87** | 0.86 | 0.85 |
| | accuracy (%) | **79.4 ± 0.2** | 78.3 ± 0.4 | 77.3 ± 0.6 |

[a] area under receiver operating characteristic curve

[b] ± shows the standard error of accuracy for each fold of cross-validation

**ml-QM-GNN**, **GNN**, and the FP-based model discussed above were then trained on those three datasets to discriminate whether a reaction is high reactive. The performance comparison for 10-fold cross validation are shown in Table S8. Two metrics are employed to evaluate model performance. Since our discriminative model generates a probability of being high reactive for a given reaction, the performance was first quantified using the area under receiver operating characteristic curve (AUC-ROC). This curve plots the true positive rate (TPR) versus the false positive rate (FPR) as the classifier tolerance is adjusted. The area under the curve thus represents a quantitative measure of the model performance, which ranges from 0.5 (random guessing) to 1.0 (perfect prediction). The AUCs for three groups of reactions through cross validation are 0.92, 0.89, and 0.87, respectively, using **ml-QM-GNN**. In addition to the ROC curve, we also calculated the prediction accuracy at the tolerance determined by the Kolmogorov-Smirnov (KS) method as a more straightforward assessment. The success rates of predicting the correct reactivity class for the three groups of reactions are then calculated to be 85.6%, 82.2%, and 79.5%, respectively. Both the ROC

and predicting accuracy indicate that the **ml-QM-GNN** model is reliable in predicting the absolute reactivity for a given reaction and provides a measurable improvement over the GNN and FP-baseline models.

### S2.5.2 Yield prediction as a regression problem

We further demonstrate the fusion ML/QM model on the absolute reactivity (yield) predictions by framing the problem as a regression task. Since the yield regression task has been demonstrated extremely challenging using text-mined reactions.[S19] Therefore, we turn to the C-N cross coupling reaction dataset of Doyle and coworkers,[S20] obtained through high-throughput experimentation. That dataset should be in higher quality than the patent data. The data set include all possible combinations of 15 reactants, four ligands, three bases, and 23 isoxazole additives in a total of 4140 reactions. We tested the **ml-QM-GNN** model on the 70/30 random splitting and 4 out-of-sample splitting with respect to the the the isoxazole additives. For both random splitting and out-of-sample splitting, our non-expert guided model achieves comparable performance to the expert-guided descriptors, but requires much less time. For detailed description of the dataset and splitting method, see ref.[S20,S21]

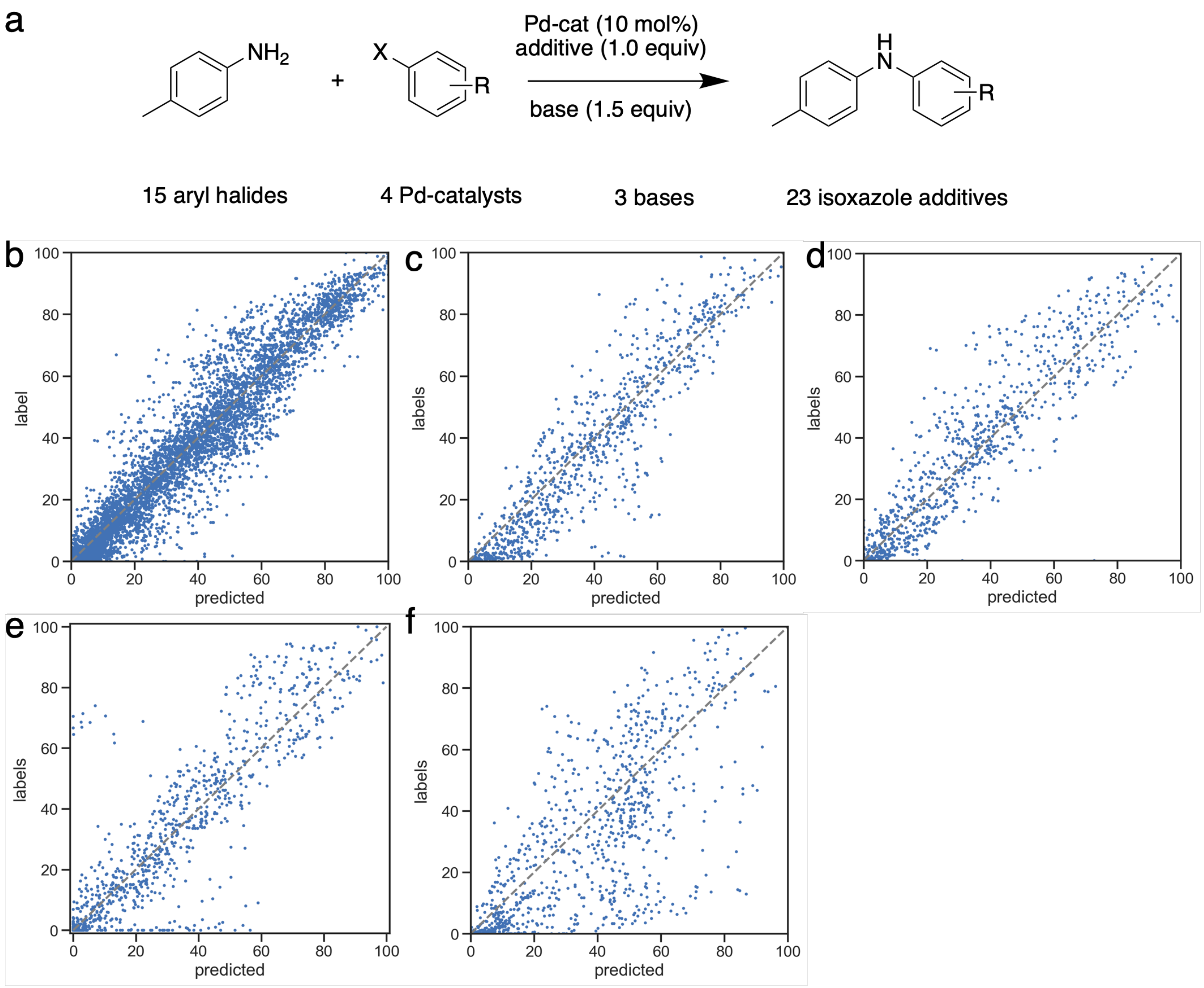


Figure S21: Prediction of yields using high-throughput experimentation data. (a) C-N cross coupling reactions by Doyle and co-workers.[S20] (b) Correlation between predicted yield and experimental yield on the 70/30 random splitting. (c)-(f) Correlation between predicted yield and experimental yield on the out-of-sample splitting with respect to isoxazole additives.

Table S9: Model comparison on the yield regression task

| $R^2$ | **random 70/30** | **OOS 1** | **OOS 2** | **OOS 3** | **OOS 4** |
|---|---|---|---|---|---|
| Doyle[a] | 0.92 | 0.80 | 0.77 | 0.64 | 0.54 |
| Glorius[b] | 0.93 | 0.85 | 0.71 | 0.64 | 0.18 |
| ml-QM-GNN | 0.90 | 0.77 | 0.81 | 0.69 | 0.29 |

[a] Feature engineering model using expert-guided descriptors[S20]
[b] Molecular fingerprint based model[S21]

## S2.6 Raw prediction accuracy for cross-validation

### S2.6.1 Raw cross-validation prediction accuracy for Figure 3A

Table S10: Raw prediction accuracy data for QM-GNN cross validation

| fold | 200 | 300 | 500 | 1000 | 2000 | all |
|---|---|---|---|---|---|---|
| fold1 | 87.0 | 89.1 | 89.7 | 89.4 | 90.9 | 91.5 |
| fold2 | 89.7 | 90.3 | 89.1 | 92.1 | 93.0 | 93.6 |
| fold3 | 85.8 | 85.2 | 85.8 | 87.3 | 86.7 | 89.4 |
| fold4 | 86.4 | 88.5 | 88.5 | 88.8 | 92.1 | 90.0 |
| fold5 | 82.7 | 86.1 | 87.6 | 88.5 | 90.9 | 90.3 |
| fold6 | 86.4 | 88.5 | 86.1 | 87.6 | 90.0 | 89.7 |
| fold7 | 87.9 | 89.1 | 89.7 | 90.0 | 91.5 | 90.6 |
| fold8 | 87.6 | 86.1 | 87.6 | 89.1 | 90.3 | 90.6 |
| fold9 | 87.6 | 90.0 | 89.1 | 91.8 | 93.1 | 93.4 |
| fold10 | 87.0 | 87.9 | 88.5 | 89.7 | 90.3 | 93.6 |

Table S11: Raw prediction accuracy data for ml-QM-GNN cross validation

| fold | 200 | 300 | 500 | 1000 | 2000 | all |
|---|---|---|---|---|---|---|
| fold1 | 87.6 | 89.1 | 90.3 | 90.9 | 90.3 | 90.0 |
| fold2 | 92.4 | 91.5 | 91.2 | 90.6 | 90.9 | 92.1 |
| fold3 | 87.3 | 86.4 | 86.1 | 86.7 | 89.4 | 89.4 |
| fold4 | 87.9 | 87.9 | 88.5 | 88.2 | 90.9 | 93.1 |
| fold5 | 85.8 | 86.4 | 86.1 | 89.7 | 87.3 | 88.2 |
| fold6 | 88.5 | 86.7 | 87.0 | 88.2 | 90.0 | 89.1 |
| fold7 | 89.1 | 87.9 | 90.0 | 88.8 | 91.2 | 90.3 |
| fold8 | 85.2 | 86.7 | 88.5 | 89.1 | 87.3 | 91.2 |
| fold9 | 90.9 | 88.8 | 90.3 | 92.1 | 93.1 | 91.8 |
| fold10 | 86.7 | 87.9 | 90.0 | 90.0 | 92.1 | 92.7 |

Table S12: Raw prediction accuracy data for GNN cross validation

| fold | 200 | 300 | 500 | 1000 | 2000 | all |
|---|---|---|---|---|---|---|
| fold1 | 78.1 | 76.0 | 82.6 | 84.4 | 88.0 | 88.9 |
| fold2 | 76.9 | 82.9 | 88.9 | 91.3 | 91.0 | 91.9 |
| fold3 | 79.3 | 81.7 | 84.7 | 86.8 | 89.8 | 91.6 |
| fold4 | 82.6 | 79.6 | 82.0 | 86.8 | 87.7 | 89.5 |
| fold5 | 83.2 | 83.2 | 86.5 | 88.9 | 91.0 | 89.5 |
| fold6 | 76.0 | 81.4 | 84.4 | 85.9 | 90.1 | 91.0 |
| fold7 | 73.3 | 81.4 | 82.3 | 85.6 | 87.1 | 86.8 |
| fold8 | 76.9 | 79.3 | 82.3 | 87.1 | 89.8 | 90.4 |
| fold9 | 75.4 | 76.6 | 83.8 | 90.1 | 88.0 | 91.9 |
| fold10 | 76.0 | 77.8 | 86.2 | 85.3 | 87.1 | 88.9 |

Table S13: Raw prediction accuracy data for QM cross validation

| fold | 200 | 300 | 500 | 1000 | 2000 | all |
|---|---|---|---|---|---|---|
| fold1 | 86.7 | 86.4 | 87.0 | 87.9 | 87.0 | 88.8 |
| fold2 | 87.0 | 84.5 | 88.8 | 89.4 | 88.2 | 87.9 |
| fold3 | 82.7 | 83.0 | 83.6 | 84.5 | 82.7 | 83.3 |
| fold4 | 87.9 | 87.3 | 87.6 | 87.9 | 88.2 | 88.5 |
| fold5 | 83.9 | 83.0 | 83.9 | 83.9 | 86.1 | 85.8 |
| fold6 | 83.3 | 83.3 | 84.5 | 86.4 | 83.9 | 85.5 |
| fold7 | 87.3 | 85.5 | 86.7 | 86.7 | 88.5 | 88.5 |
| fold8 | 87.0 | 87.0 | 86.7 | 86.7 | 86.7 | 88.5 |
| fold9 | 90.0 | 89.7 | 89.4 | 90.3 | 90.0 | 91.2 |
| fold10 | 84.2 | 85.8 | 86.4 | 84.8 | 86.1 | 86.1 |

#### S2.6.2 Raw cross-validation prediction accuracy for Figure 7A

Table S14: Raw prediction accuracy data for ml-QM-GNN cross validation

| fold | 300[a] | | | 500 | | | 1000 | | | 2000 | | | 3000 | | | 5000 | | |
|---|---|---|---|---|---|---|---|---|---|---|---|---|---|---|---|---|---|---|
| | 1[b] | 2 | 3 | 1 | 2 | 3 | 1 | 2 | 3 | 1 | 2 | 3 | 1 | 2 | 3 | 1 | 2 | 3 |
| fold0 | 83.1 | 91.3 | 93.5 | 84.3 | 93.1 | 93.7 | 85.0 | 92.9 | 94.2 | 86.3 | 92.9 | 94.9 | 88.8 | 94.9 | 95.1 | 88.1 | 94.9 | 95.9 |
| fold1 | 81.4 | 88.3 | 94.9 | 82.1 | 91.9 | 94.6 | 83.5 | 93.9 | 96.4 | 84.3 | 94.0 | 97.5 | 85.6 | 93.2 | 98.1 | 90.7 | 95.6 | 98.7 |
| fold2 | 85.2 | 88.9 | 93.4 | 85.8 | 91.5 | 96.4 | 87.7 | 94.7 | 96.5 | 86.4 | 95.6 | 96.2 | 87.0 | 96.7 | 97.0 | 89.7 | 97.3 | 97.8 |
| fold3 | 85.1 | 89.3 | 95.1 | 86.0 | 89.7 | 95.9 | 86.3 | 92.9 | 96.5 | 86.6 | 93.3 | 96.5 | 86.6 | 94.1 | 97.9 | 89.8 | 95.6 | 98.3 |
| fold4 | 82.2 | 88.5 | 93.5 | 83.9 | 91.2 | 93.7 | 84.5 | 91.3 | 96.5 | 85.8 | 94.7 | 97.6 | 85.2 | 95.9 | 97.3 | 87.1 | 96.4 | 97.9 |
| fold5 | 84.8 | 90.7 | 96.7 | 84.7 | 91.1 | 95.7 | 85.3 | 92.3 | 96.4 | 86.6 | 94.0 | 97.6 | 87.9 | 95.6 | 97.5 | 89.8 | 95.9 | 98.3 |
| fold6 | 83.7 | 90.9 | 95.4 | 85.1 | 90.5 | 96.8 | 86.2 | 92.9 | 97.0 | 86.6 | 94.3 | 96.4 | 86.4 | 94.7 | 97.3 | 91.2 | 95.9 | 97.3 |
| fold7 | 85.6 | 91.6 | 93.8 | 85.1 | 92.9 | 93.8 | 87.1 | 94.4 | 95.7 | 90.0 | 95.5 | 96.4 | 90.0 | 97.3 | 96.4 | 90.8 | 97.5 | 97.5 |
| fold8 | 84.1 | 89.5 | 95.3 | 84.1 | 92.1 | 95.4 | 85.6 | 93.7 | 95.4 | 87.7 | 94.4 | 96.5 | 90.7 | 95.9 | 95.4 | 87.5 | 96.7 | 97.0 |
| fold9 | 87.3 | 90.8 | 94.5 | 87.9 | 91.7 | 94.6 | 88.3 | 94.7 | 95.7 | 89.7 | 94.7 | 95.9 | 89.6 | 95.7 | 95.9 | 91.7 | 95.1 | 97.0 |

[a] Training size

[b] Reaction sub-class

Table S15: Raw prediction accuracy data for GNN-baseline cross validation

| fold | 300 | | | 500 | | | 1000 | | | 2000 | | | 3000 | | | 5000 | | |
|---|---|---|---|---|---|---|---|---|---|---|---|---|---|---|---|---|---|---|
| | 1 | 2 | 3 | 1 | 2 | 3 | 1 | 2 | 3 | 1 | 2 | 3 | 1 | 2 | 3 | 1 | 2 | 3 |
| fold0 | 73.7 | 91.5 | 92.1 | 76.6 | 90.9 | 93.7 | 78.3 | 92.7 | 95.4 | 83.7 | 91.5 | 94.8 | 85.4 | 94.4 | 96.2 | 87.3 | 94.3 | 94.9 |
| fold1 | 70.5 | 87.7 | 96.7 | 72.6 | 89.3 | 96.5 | 78.2 | 91.3 | 97.3 | 84.8 | 91.5 | 96.8 | 83.9 | 92.9 | 96.7 | 92.5 | 94.7 | 97.9 |
| fold2 | 74.7 | 88.8 | 94.9 | 77.4 | 89.7 | 94.9 | 79.9 | 91.1 | 96.5 | 83.1 | 92.3 | 96.8 | 84.6 | 95.7 | 97.0 | 85.9 | 96.0 | 97.5 |
| fold3 | 75.2 | 87.4 | 91.0 | 73.8 | 89.5 | 93.2 | 76.2 | 90.8 | 94.8 | 85.0 | 93.1 | 95.3 | 85.9 | 93.7 | 96.1 | 89.6 | 94.5 | 96.8 |
| fold4 | 68.5 | 86.4 | 93.7 | 77.2 | 87.7 | 96.1 | 79.1 | 90.0 | 95.7 | 82.2 | 93.3 | 96.5 | 86.6 | 93.6 | 96.2 | 87.4 | 93.7 | 97.5 |
| fold5 | 73.0 | 90.1 | 94.0 | 73.7 | 90.8 | 95.7 | 81.0 | 90.9 | 97.8 | 82.8 | 92.5 | 96.7 | 85.9 | 93.5 | 96.4 | 87.9 | 93.6 | 97.2 |
| fold6 | 76.8 | 86.4 | 96.2 | 78.9 | 87.9 | 94.3 | 82.2 | 90.4 | 95.6 | 83.1 | 93.3 | 94.6 | 85.1 | 93.5 | 95.9 | 89.0 | 94.5 | 97.2 |
| fold7 | 76.4 | 87.9 | 93.4 | 80.4 | 87.7 | 94.2 | 81.2 | 92.1 | 95.3 | 84.0 | 95.3 | 95.4 | 85.8 | 96.0 | 95.6 | 91.1 | 96.5 | 95.7 |
| fold8 | 72.2 | 86.7 | 89.6 | 75.5 | 89.3 | 95.6 | 79.0 | 90.9 | 95.4 | 86.0 | 92.3 | 97.0 | 87.0 | 94.5 | 96.5 | 90.9 | 95.3 | 97.5 |
| fold9 | 74.1 | 87.5 | 91.6 | 79.4 | 89.3 | 91.6 | 85.1 | 91.2 | 95.1 | 85.2 | 92.3 | 94.6 | 87.4 | 93.2 | 94.9 | 89.7 | 94.4 | 96.8 |

Table S16: Raw prediction accuracy data for FP-baseline cross validation

| fold | 300 | | | 500 | | | 1000 | | | 2000 | | | 3000 | | | 5000 | | |
|---|---|---|---|---|---|---|---|---|---|---|---|---|---|---|---|---|---|---|
| | 1 | 2 | 3 | 1 | 2 | 3 | 1 | 2 | 3 | 1 | 2 | 3 | 1 | 2 | 3 | 1 | 2 | 3 |
| fold0 | 65.6 | 83.4 | 90.0 | 71.3 | 86.6 | 90.4 | 74.7 | 91.1 | 89.4 | 76.8 | 94.0 | 94.3 | 81.7 | 95.1 | 94.2 | 84.3 | 94.9 | 95.4 |
| fold1 | 64.0 | 81.5 | 90.2 | 64.9 | 86.7 | 91.9 | 74.7 | 88.4 | 92.3 | 74.7 | 92.0 | 93.8 | 80.4 | 93.3 | 95.4 | 83.2 | 94.7 | 95.3 |
| fold2 | 63.3 | 78.3 | 87.5 | 72.2 | 83.1 | 91.3 | 74.3 | 87.7 | 92.1 | 82.8 | 91.3 | 92.1 | 78.6 | 92.0 | 94.2 | 82.2 | 93.6 | 95.1 |
| fold3 | 66.0 | 81.0 | 88.5 | 71.0 | 87.4 | 92.4 | 74.9 | 89.1 | 92.1 | 76.4 | 92.0 | 95.9 | 81.2 | 92.5 | 96.8 | 86.0 | 94.4 | 96.1 |
| fold4 | 63.9 | 79.5 | 91.3 | 69.1 | 85.8 | 91.6 | 70.4 | 88.4 | 92.4 | 77.5 | 89.2 | 93.8 | 76.8 | 91.3 | 95.4 | 82.2 | 92.0 | 95.6 |
| fold5 | 60.0 | 83.0 | 88.3 | 68.7 | 82.3 | 91.1 | 76.4 | 86.7 | 92.6 | 77.2 | 90.4 | 93.7 | 84.5 | 90.9 | 94.9 | 87.5 | 91.9 | 95.4 |
| fold6 | 64.9 | 83.1 | 89.7 | 69.2 | 86.6 | 92.9 | 75.5 | 89.6 | 93.4 | 81.2 | 92.5 | 93.7 | 80.1 | 93.9 | 95.1 | 83.1 | 93.7 | 96.8 |
| fold7 | 66.5 | 85.4 | 87.7 | 75.5 | 88.0 | 91.6 | 76.4 | 87.1 | 91.6 | 78.2 | 91.5 | 93.5 | 82.2 | 94.1 | 94.5 | 84.4 | 96.0 | 94.9 |
| fold8 | 65.2 | 80.0 | 87.5 | 70.6 | 85.5 | 91.9 | 76.6 | 85.8 | 92.9 | 81.2 | 90.9 | 94.5 | 78.0 | 91.6 | 95.1 | 83.9 | 92.5 | 96.5 |
| fold9 | 67.9 | 83.2 | 87.7 | 72.2 | 86.3 | 90.0 | 77.2 | 89.2 | 92.6 | 84.3 | 90.1 | 94.6 | 80.5 | 92.5 | 95.9 | 83.3 | 94.1 | 96.5 |

# References


(S1) Pistachio (NextMove Software). `https://www.nextmovesoftware.com/pistachio.html`.

(S2) Coley, C. W.; Green, W. H.; Jensen, K. F. RDChiral: An RDKit wrapper for handling stereochemistry in retrosynthetic template extraction and application. *Journal of chemical information and modeling* **2019**, *59*, 2529–2537.

(S3) Hirshfeld, F. L. Bonded-atom fragments for describing molecular charge densities. *Theoretica chimica acta* **1977**, *44*, 129–138.

(S4) Wiberg, K. B.; Rablen, P. R. Comparison of atomic charges derived via different procedures. *Journal of Computational Chemistry* **1993**, *14*, 1504–1518.

(S5) Fuentealba, P.; Pérez, P.; Contreras, R. On the condensed Fukui function. *The Journal of Chemical Physics* **2000**, *113*, 2544–2551.

(S6) Yang, W.; Mortier, W. J. The use of global and local molecular parameters for the analysis of the gas-phase basicity of amines. *Journal of the American Chemical Society* **1986**, *108*, 5708–5711.

(S7) Roy, R. K.; Pal, S.; Hirao, K. On non-negativity of Fukui function indices. *The Journal of chemical physics* **1999**, *110*, 8236–8245.

(S8) Verma, R. P.; Hansch, C. Use of 13C NMR chemical shift as QSAR/QSPR descriptor. *Chemical reviews* **2011**, *111*, 2865–2899.

(S9) Wolinski, K.; Hinton, J. F.; Pulay, P. Efficient implementation of the gauge-independent atomic orbital method for NMR chemical shift calculations. *Journal of the American Chemical Society* **1990**, *112*, 8251–8260.

(S10) Riniker, S.; Landrum, G. A. Better informed distance geometry: using what we know to improve conformation generation. *Journal of chemical information and modeling* **2015**, *55*, 2562–2574.

(S11) Halgren, T. A. Merck molecular force field. I. Basis, form, scope, parameterization, and performance of MMFF94. *Journal of computational chemistry* **1996**, *17*, 490–519.

(S12) Bannwarth, C.; Ehlert, S.; Grimme, S. GFN2-xTB—An accurate and broadly parametrized self-consistent tight-binding quantum chemical method with multipole electrostatics and density-dependent dispersion contributions. *Journal of chemical theory and computation* **2019**, *15*, 1652–1671.

(S13) Shervashidze, N.; Schweitzer, P.; Van Leeuwen, E. J.; Mehlhorn, K.; Borgwardt, K. M. Weisfeiler-lehman graph kernels. *Journal of Machine Learning Research* **2011**, *12*.

(S14) Yang, K.; Swanson, K.; Jin, W.; Coley, C.; Eiden, P.; Gao, H.; Guzman-Perez, A.; Hopper, T.; Kelley, B.; Mathea, M., et al. Analyzing learned molecular representations for property prediction. *Journal of chemical information and modeling* **2019**, *59*, 3370–3388.

(S15) Bemis, G. W.; Murcko, M. A. The properties of known drugs. 1. Molecular frameworks. *Journal of medicinal chemistry* **1996**, *39*, 2887–2893.

(S16) Wu, Y.; Liu, L.; Bae, J.; Chow, K.-H.; Iyengar, A.; Pu, C.; Wei, W.; Yu, L.; Zhang, Q. Demystifying Learning Rate Policies for High Accuracy Training of Deep Neural Networks. 2019 IEEE International Conference on Big Data (Big Data). 2019; pp 1971–1980.

(S17) Heid, E.; Fleck, M.; Chatterjee, P.; Schröder, C.; MacKerell Jr, A. D. Toward Prediction of Electrostatic Parameters for Force Fields That Explicitly Treat Electronic Polarization. *Journal of chemical theory and computation* **2019**, *15*, 2460–2469.

(S18) Skoraczyński, G.; Dittwald, P.; Miasojedow, B.; Szymkuć, S.; Gajewska, E.; Grzybowski, B. A.; Gambin, A. Predicting the outcomes of organic reactions via machine learning: are current descriptors sufficient? *Scientific reports* **2017**, *7*, 1–9.

(S19) Schwaller, P.; Vaucher, A. C.; Laino, T.; Reymond, J.-L. Prediction of Chemical Reaction Yields using Deep Learning. 2020; https://chemrxiv.org/articles/preprint/Prediction_of_Chemical_Reaction_Yields_using_Deep_Learning/12758474/1.

(S20) Ahneman, D. T.; Estrada, J. G.; Lin, S.; Dreher, S. D.; Doyle, A. G. Predicting reaction performance in C–N cross-coupling using machine learning. *Science* **2018**, *360*, 186–190.

(S21) Sandfort, F.; Strieth-Kalthoff, F.; Kühnemund, M.; Beecks, C.; Glorius, F. A Structure-Based Platform for Predicting Chemical Reactivity. *Chem* **2020**, *6*, 1379 – 1390.
